# Magnetic phase diagram of the solid solution LaMn_2_(Ge_1−*x*_Si_*x*_)_2_ (0 ≤ *x* ≤ 1) unraveled by powder neutron diffraction

**DOI:** 10.1038/s41598-022-12549-y

**Published:** 2022-06-03

**Authors:** Stefanie Siebeneichler, Alexander Ovchinnikov, Brianna Bosch-Santos, Gabriel A. Cabrera-Pasca, Roxana Flacau, Qingzhen Huang, Artur W. Carbonari, Dominic Ryan, Anja-Verena Mudring

**Affiliations:** 1 Department of Materials and Environmental Chemistry, Stockholm University, Svante Arrhenius väg 16 C, 10691 Stockholm, Sweden; 2 Material Measurement Laboratory, National Institute of Standards and Technology-NIST, Gaithersburg, MD 20899 USA; 3 Instituto de Pesquisas Energéticas e Nucleares–IPEN-CNEN/SP, São Paulo, SP 05508-000 Brazil; 4 Programa de Pós-Graduação em Ciência e Engenharia de Materiais–PPGCEM, Universidade Federal do Pará, Ananindeua, PA 67130 660 Brazil; 5 Canadian Neutron Beam Centre, Chalk River Laboratories, Chalk River, ON K0J 1J0 Canada; 6 Center for Neutron Research, National Institute of Standards and Technology, Gaithersburg, MD 20899 USA; 7 The Centre for the Physics of Materials and the Physics Department, McGill University, 3600 University St., Montreal, QC H3A 2T8 Canada; 8 Department of Chemistry, Aarhus University, Langelandsgade 140, 8000 Aarhus C, Denmark

**Keywords:** Inorganic chemistry, Materials chemistry, Magnetic materials

## Abstract

The structural and magnetic properties of the ThCr_2_Si_2_-type solid solution LaMn_2_(Ge_1−*x*_Si_*x*_)_2_ (*x* = 0.0 to 1.0) have been investigated employing a combination of X-ray diffraction, magnetization and neutron diffraction measurements, which allowed establishing a magnetic composition-temperature phase diagram. Substitution of Ge by Si leads to a compression of the unit cell, which affects the magnetic exchange interactions. In particular, the magnetic structure of LaMn_2_(Ge_1−*x*_Si_*x*_)_2_ is strongly affected by the unit cell parameter *c*, which is related to the distance between adjacent Mn layers. Commensurate antiferromagnetic layers and a canted ferromagnetic structure dominate the Si-rich part of the solid solution, whilst an incommensurate antiferromagnetic flat spiral and a conical magnetic structure are observed in the Si-poor part.

## Introduction

**Figure 1:**
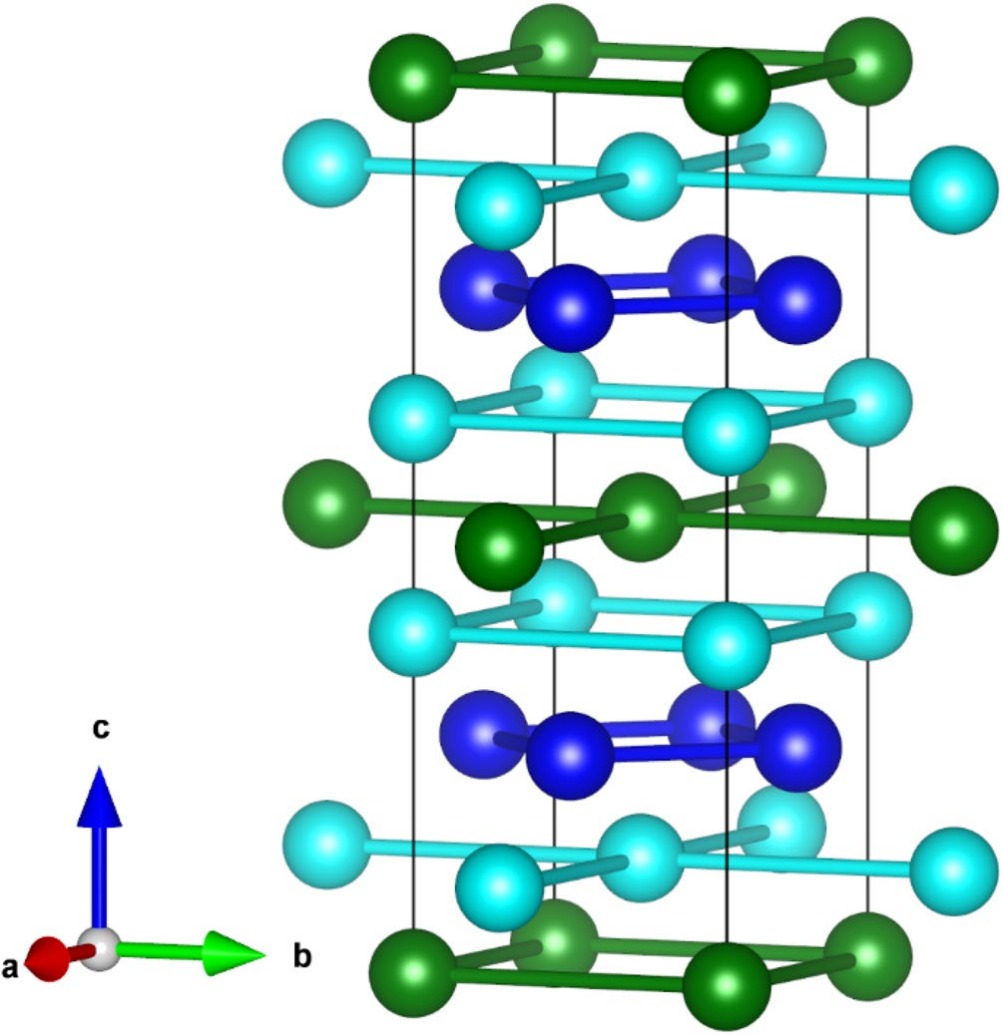
Crystal structure of LaMn_2_(Ge_1-*x*_Si_*x*_)_2_: La (*green*), Mn (*dark blue*), Si/Ge (*turquoise*).

Materials belonging to the *AM*_2_*X*_2_ (*A* = alkali, alkaline-earth, or a rare-earth element, *M* = transition metal, *X* = a main group element) family of compounds are known to show a wide spectrum of intriguing physical phenomena including magnetism, superconductivity, heavy fermions, quantum critical points and Kondo behavior^1–4^. Its members preferentially crystallize in the ThCr_2_Si_2_-type structure (space group *I*4/*mmm*) in which the *A*, *M* and *X* atoms occupy the 2*a*, 4*d* and 4*e* crystallographic sites, respectively. This atomic arrangement leads each of the three elements to form square nets stacked along the crystallographic *c* axis in the order *A*-*X*-*M*-*X*-*A*, *c.f.* Figure 1. The structure can also be described as being composed of layers of edge-sharing *XM*_4_ square pyramids of alternating orientation between square nets of *A* atoms. A third structure description is as layers of edge-sharing *MX*_4_ tetrahedra alternating with square nets of the *A* metal. Illustrations of the latter two crystal structure description can be found in the Supplementary Fig. S1.

The subgroup of manganese silicides and germanides, *RE*Mn_2_*X*_2_ (*RE* = rare-earth metal, *X* = Si, Ge) has gained particular attention for their interesting physical properties. Giant magnetoresistance (GMR) was observed in *RE*Mn_2_Ge_2_ (*RE* = La, Sm)^5–7^, magnetocaloric behavior in *RE*Mn_2_Si_2_ (*RE* = Ho, Er, Tb)^8–10^ and *RE*Mn_2_Ge_2_ (*RE* = Ce, Tb)^11,12^ and skyrmionic bubbles in *RE*Mn_2_Ge_2_ (*RE* = Ce, Pr, Nd)^13^. Recently, LaMn_2_Ge_2_ has been shown to demonstrate a topological Hall effect (THE)^14^.

The wide range of behavior exhibited by the *RE*Mn_2_*X*_2_ materials is related to the rich diversity of collinear and non-collinear magnetic states that can be realized in this atomic arrangement^15–26^. Magnetic ordering temperatures between 300 and 714 K have been observed for all compounds of this type^1,27^, and many of them undergo several magnetic transitions upon cooling before they reach their magnetic ground state. Previous studies on various solid solutions based on *RE*Mn_2_*X*_2_ indicated that the Mn–Mn distances play a major role in the formation of different magnetic structures. At intra-planar nearest-neighbor Mn–Mn distances *d*_intra_ < 2.84 Å, the magnetic moments within the Mn-layers order in a ferromagnetic out-of-plane arrangement while adjacent square nets couple antiferromagnetically. When *d*_intra_ surpasses the critical distance of 2.84 Å a transition from the ferromagnetic out-of-plane arrangement to an antiferromagnetic in-plane one takes place. At the same time, the coupling between the planes remains antiferromagnetic^11,28–30^. A further increase to *d*_intra_ > 2.87 Å results in a second transition in which the intra-layer arrangement remains unchanged but the inter-layer coupling evolves from antiferromagnetic to ferromagnetic^11,28–31^.

The ternaries LaMn_2_*X*_2_ (*X* = Si, Ge) both exhibit magnetic structures with ferromagnetic inter-layer coupling. Adopting the nomenclature commonly used in the literature (the detailed description follows below)^11,28,29,31–33^, LaMn_2_Si_2_ exhibits a magnetic structure referred to as antiferromagnetic layers (AFl) below 470 K^15,34,35^ and undergoes a transition to the ferromagnetic mixed commensurate (Fmc) structure at 310 K on cooling^15,35,36^. Additional incommensurate modulation peaks occur below 40 K and remain down to low temperatures, which was interpreted as co-existence of the ferromagnetic mixed incommensurate (Fmi) and ferromagnetic mixed commensurate (Fmc) structures^15,35^. In contrast, LaMn_2_Ge_2_ is reported to adopt the antiferromagnetic flat spiral (AFfs) below 425 K. On cooling, a transition into the Fmi structure occurs at *T*_C_ = 325 K, and this state is preserved down to 2 K^15,28,34,36^.

The Fmc and Fmi structures observed in LaMn_2_Si_2_ and LaMn_2_Ge_2_, respectively, share some similarities: Both exhibit a ferromagnetic out-of-plane magnetic moment component as well as an identical checkerboard in-plane spin arrangement within the Mn nets. The incommensurability for the Fmi structure of LaMn_2_Ge_2_ results from a rotation of the magnetic moments in the adjacent square nets with respect to each other along the tetragonal *c* axis. In the case of the commensurate Fmc structure observed for LaMn_2_Si_2_, the magnetic moments in neighboring planes are rotated by 180° along *c*. Previous studies on the solid solution LaMn_2_(Si_1−*x*_Ge_*x*_)_2_ (*x* = 0, 0.2, 0.4, 0.6, 0.8, 1) using Perturbed Angular Correlation (PAC) spectroscopy with ^111^In(^111^Cd) and ^140^La(^140^Ce) as probe nuclei have shown that the Curie and Neél temperatures in both ternaries can be tuned by Si/Ge mixing^34,36^. This indicates that different compositions of the solid solution LaMn_2_(Si_1−*x*_Ge_*x*_)_2_ will exhibit magnetic behavior similar to that of the ternary compounds and that the magnetic transition temperatures can be tuned by the composition. Thus, a study of the influence of the amount of Si/Ge mixing on the magnetic incommensurability and co-existing magnetic phases becomes necessary. Furthermore, the physical properties relevant for potential application of the LaMn_2_*X*_2_ compounds (*X* = Si, Ge) are directly linked to their magnetism. For this purpose, we investigated the effect of the substitution of Ge by Si in the solid solution LaMn_2_(Ge_1−*x*_Si_*x*_)_2_ on the structural and magnetic properties by magnetization, X-ray and neutron diffraction measurements.

## Results

### Powder X-ray diffraction measurements

**Figure 2:**
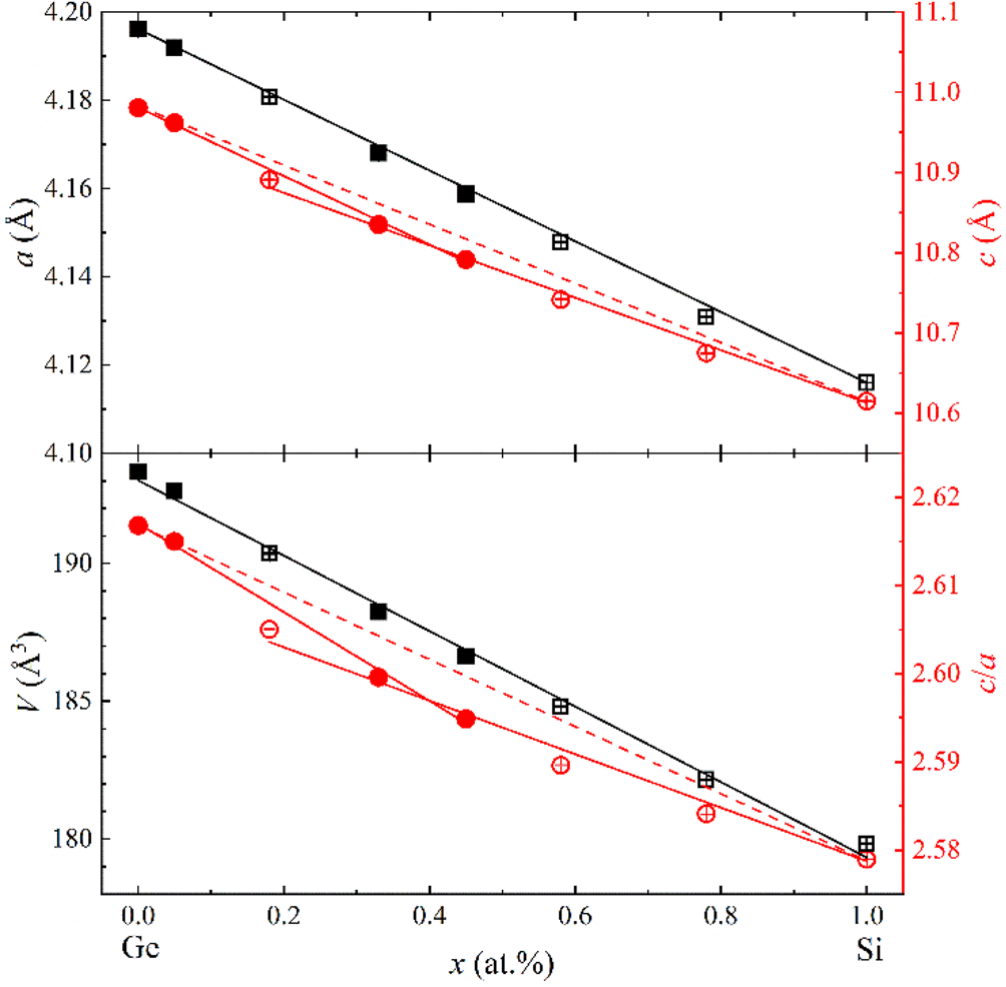
Composition dependence of the LaMn_2_(Ge_1–*x*_Si_*x*_)_2_ lattice parameters *a* and *c* (*top*), cell ratio *c*/*a* and unit cell volume *V* (*bottom*) at room temperature. The data points of the samples synthesized by furnace annealing (*x* = 0, 0.05, 0.33, 0.47) are highlighted by *filled symbols*, the ones prepared by arc melting (*x* = 0.18, 0.58, 0.78, 1) by *empty symbols*. The compression of *a* follows Vegard’s law (*top*, *solid lines*), but *c* deviates from this linear trend (*top*, *dashed line*). The same anomalous behavior is also observable in the cell ratio *c*/*a* (*bottom*). In order to compensate possible errors from the different sample preparation techniques, the *c* and *c/a* values of the samples prepared by arc melting and solid state synthesis were fitted separately. All error bars are shown and represent 1σ. However, the error bars may be smaller than the symbol.

Rietveld refinements of the PXRD data indicate that all samples of LaMn_2_(Ge_1−*x*_Si_*x*_)_2_ crystallize in the ThCr_2_Si_2_-type structure. The lattice parameters *a* and *c* of the ternary compounds are in good agreement with the values reported in the literature^15^. Table 1 lists the lattice parameters of all samples. The quaternary samples are labelled according to their refined compositions. The composition dependence of *a*, *c*, the cell ratio *c*/*a* and the unit cell volume *V* is plotted in Fig. 2. The partial substitution of Ge by Si leads to a compression of lattice parameters at room temperature. The lattice parameter *a* follows Vegard’s law^37^ (Fig. 2 top) over the whole composition range. In contrast, the *c* parameter exhibits deviations from a linear behavior (*dashed lines*). A steeper decline of *c* is observed in the Ge-rich part of LaMn_2_(Ge_1−*x*_Si_*x*_)_2_. This anomaly is also reflected in the behavior of the *c*/*a* ratio (Fig. 2 bottom). The change in slope of *c* is correlated to the magnetic properties, vide infra. The *z* component of the crystallographic site of Si/Ge (0, 0, *z*) remains at a nearly constant value of about 0.38 throughout the whole solid solution.

**Table 1:** Lattice parameters *a* and *c*, the cell ratio *c*/*a* and the unit cell volume *V* of LaMn_2_(Ge_1-*x*_Si_*x*_)_2_ (*x* = 0, 0.05, 0.18, 0.33, 0.47, 0.58, 0.78, 1) from refined PXRD data.

Compound	*a* (Å)	*c* (Å)	*c*/*a*	*V* (Å^3^)	*T*_C_^c^ (K)	*M*_sat_^c^ (µ_B_/Mn)
*x* = 0^a^	4.19619(3)	10.98074(8)	2.61684(2)	193.349(6)	326.22(3)	1.3005(1)
*x* = 0.05^a^	4.19198(2)	10.96205(8)	2.61501(2)	192.633(4)	324.68(3)	1.4317(2)
*x* = 0.18	4.18070(8)	10.8909(2)	2.60503(7)	190.35(2)	318.92(3)	1.4663(2)
*x* = 0.33^a^	4.16804(4)	10.8352(1)	2.59960(4)	188.235(8)	319.35(2)	1.2678(4)
*x* = 0.47^a^	4.15880(4)	10.7915(1)	2.59486(3)	186.646(8)	319.09(3)	1.2492(2)
*x* = 0.58^a^	4.14792(9)	10.7415(2)	2.58962(8)	184.81(2)	316.28(2)	^b^
*x* = 0.78	4.13098(6)	10.6749(1)	2.58411(5)	182.17(1)	311.54(4)	^b^
*x* = 1	4.11599(2)	10.61495(6)	2.57895(2)	179.832(4)	308.53(3)	^b^

The Curie temperature *T*_C_ and approximate values for the saturation magnetization *M*_sat_^b^ at 2 K and 6 T were determined from the magnetization measurements. The numbers between parentheses show the error bars and represent 1σ.

^a^Sample contains minor impurity, see “Methods” and Supplementary Information.

^b^No saturation observed for *x* = 0.58, 0.78, 1 under the maximum applied fields.

^c^The standard deviations are calculated from the fits. The experimental errors for *M*_sat_ are estimated to be of the order of 10^−3^ µ_B_/Mn.

### Magnetization measurements

**Figure 3:**
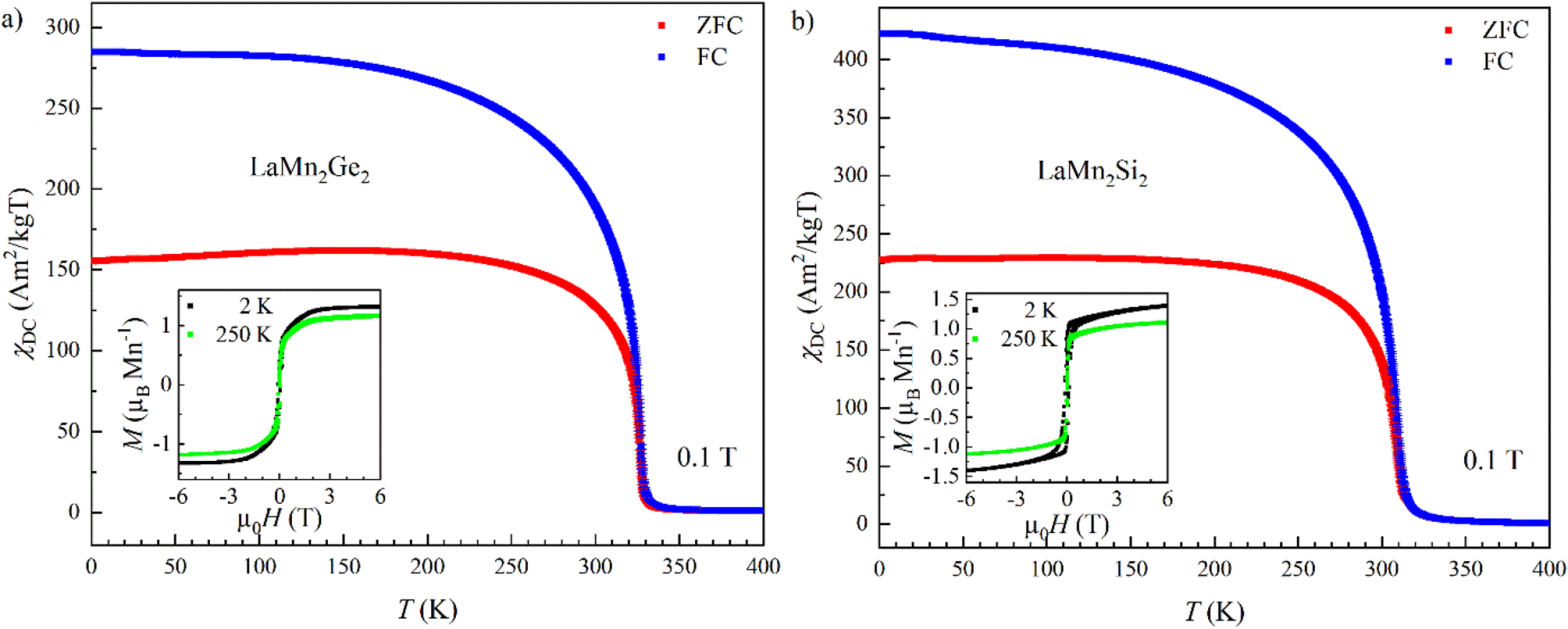
Zero field-cooled (ZFC, *red*) and field-cooled (FC, *blue*) magnetic susceptibilities and isothermal magnetization (*insets*) of LaMn_2_Ge_2_ (**a**) and LaMn_2_Si_2_ (**b**). All error bars are shown and represent 1σ. However, the error bars may be smaller than the symbol.

Temperature dependence of the DC magnetic susceptibility *χ*_DC_ for ternary LaMn_2_Ge_2_ and LaMn_2_Si_2_ between 2 and 400 K is plotted in Fig. 3. Both compounds undergo a ferromagnetic-like transition at their respective Curie temperatures *T*_C_ = 326.22(3) K and 308.53(3) K. Above *T*_C_, the inverse susceptibilities *χ*_*DC*_^*−1*^(*T*) do not follow a linear behavior (not shown) up to 400 K and suggest that LaMn_2_Ge_2_ and LaMn_2_Si_2_ do not fully enter the paramagnetic regime up to the highest measured temperature. This is also supported by the powder neutron diffraction data presented below. Similar behavior of the DC magnetic susceptibility was also observed for the quaternary samples (Supplementary Fig. S3). *T*_C_ decreases from LaMn_2_Ge_2_ to LaMn_2_Si_2_ as a function of the Ge/Si mixing (Table 1). The Curie temperatures of all samples were determined by fitting the first derivative of the susceptibility d*χ*_*DC*_/d*T* with a Gaussian peak function (Supplementary Fig. S4a,b). The transition temperatures extracted from our data are in fair agreement with the literature^36^. Thus, the *T*_C_ values reported in previous studies go from 323.3(2) K in LaMn_2_Ge_2_ to 308.5(2) K in LaMn_2_Si_2_.

The isothermal magnetization curves of the ternary LaMn_2_Ge_2_ and LaMn_2_Si_2_ measured at 2 K and 250 K between − 6 and 6 T exhibit typical ferromagnetic behavior (Fig. 3 insets). For LaMn_2_Ge_2_, the *M*(*H*) curve reaches *M*_sat_ = 1.3005(1) µ_B_/Mn at 2 K and *M*_sat_ = 1.0976(1) µ_B_/Mn at 250 K. In contrast, the *M*(*H*) curve of LaMn_2_Si_2_ shows no saturation under the same applied magnetic fields at 2 K, but reaches saturation with *M*_sat_ = 0.99823(4) µ_B_/Mn at 250 K. A similar behavior to LaMn_2_Si_2_ is also observed for the Si-rich quaternary samples with the composition *x* = 0.58 and 0.78 (Supplementary Fig. S3): They reach saturation at 250 K, but not at 2 K. This lack of saturation possibly indicates the presence of an antiferromagnetic component^38^. Furthermore, the low saturation magnetization observed for all compositions points toward a magnetic structure different from that of a simple ferromagnet, which will be discussed below. Hysteresis loops were observed for all compositions. Supplementary Fig. S5a,b highlight the isothermal magnetization of all samples between − 1 and 1 T. On raising the temperature from 2 to 250 K, the coercive field *H*_c_ of the samples with compositions *x* = 0.33, 0.47, 0.58, 0.78 and 1 decreases clearly whereas *H*_c_ remains nearly constant for *x* = 0, 0.05 and 0.18 (Supplementary Table S1). The change of the coercive field on warming hints at a magnetic phase transition occurring between 2 and 250 K which is also confirmed by the PND data presented below. The different coercive fields between samples are attributed to variations in particle size^39^. The magnetization data observed here are in line with the literature^15,38,40,41^. As the samples only contain small amounts of impurities, we believe the impurities do not affect the magnetization data in a significant way.

### Powder neutron diffraction measurements

**Figure 4:**
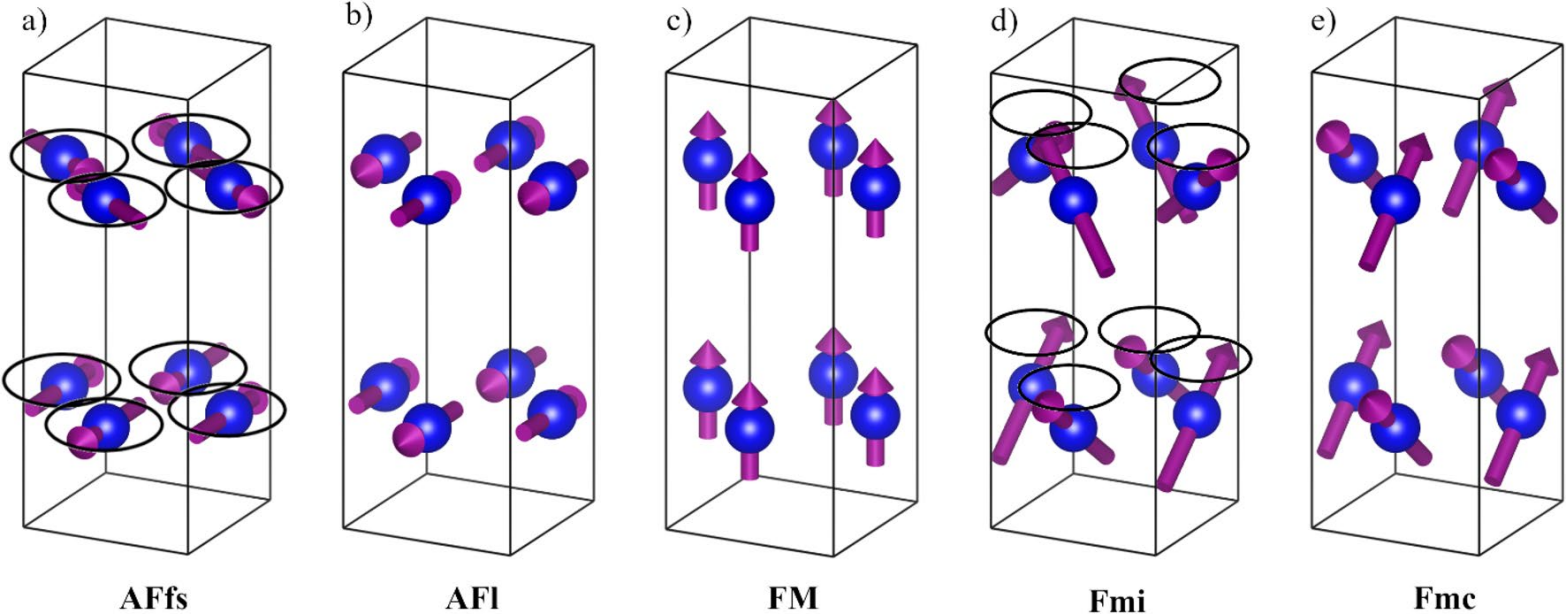
Models for the spin arrangements of the five diffraction conditions observed in LaMn_2_(Ge_1−*x*_Si_*x*_)_2_: (**a**) antiferromagnetic flat spiral (AFfs), (**b**) antiferromagnetic layers (AFl), (**c**) ferromagnetic contribution (FM), (**d**) ferromagnetic mixed incommensurate (Fmi) and (**e**) ferromagnetic mixed commensurate (Fmc). The circles in (**a**) and (**d**) indicate the full rotation of the spins along (**c**) in the incommensurate AFfs and Fmi. FM does not exist as an independent phase but contributes to Fmi and Fmc.

Powder neutron diffraction (PND) patterns were collected for a series of samples with the composition LaMn_2_(Ge_1−*x*_Si_*x*_)_2_ (*x* = 0, 0.05, 0.18, 0.33, 0.47, 0.58, 0.78, 1). Following previous works^11,28,29,31–33,42^, we will describe the observed magnetic structures based on elementary magnetic components. The latter can be readily identified from characteristic magnetic reflections:

1. The antiferromagnetic flat spiral (AFfs) can be described as an antiferromagnetic alignment of magnetic moments within the square lattice for each Mn layer. The spin motive of each layers is the same but the moments are rotated by an angle *φ* in adjacent layers, by 2*φ* in case of the next-nearest layer and so on. Therefore, the magnetic moments in AFfs form a flat spiral along the *c*-axis. (Fig. 4a) The incommensurate propagation vector (0, 0, *k*_z_) describes the length of the spiral or how many crystal unit cells are necessary until the magnetic moments have reached a full rotation. In the diffraction patterns, the AFfs can be identified by pairs of low intensity, magnetic modulation peaks appearing around reflections with the diffraction condition *h* + *k* = 2*n* + 1: e.g. satellite reflections (101)^−^/(101)^+^ and (103)^−^/(103)^+^ around (101) and (103), respectively.
2. The structure of antiferromagnetic layers (AFl) consists of the same antiferromagnetic arrangement of magnetic moments within the square lattice as in the case of AFfs. However, the moments in adjacent layers are rotated by 180° along *c*. (Fig. 4b) The magnetic reflections of AFl can be indexed with a *k*-vector of (0, 0, 0) and add intensity to nuclear Bragg peaks with the reflection condition *h* + *k* = 2*n* + 1. The magnetic signal of the AFl contribution is especially visible for (101) and (103).
3. In the ferromagnetic (FM) component, all magnetic moments are aligned along *c* (Fig. 4c). The FM contribution is found on nuclear Bragg peaks fulfilling the reflection conditions *h* + *k* = 2*n* and *l* = 2*n*. Therefore, the FM Bragg peaks increase the intensity of nuclear Bragg peaks. This is most noticeable for the reflections (002) and (112).

Before presenting the results of the PND studies, we would like to provide some general comments about the magnetic structures:

Reflection condition (1) points to incommensurate magnetic modulation (IC), while conditions (2) and (3) indicate commensurate magnetic reflections (C). The Bragg markers corresponding to the magnetic phases in the PND patterns presented below are separated into IC and C contributions. The sets of magnetic peaks corresponding to either of the magnetic components (1) or (2) can be observed in PND patterns in the absence of other magnetic reflections, suggesting that these two elementary components represent actual magnetic structures. In addition, more complex magnetic arrangements result from combinations of the elementary contributions listed above:

4. The ferromagnetic mixed incommensurate structure (Fmi) is a superposition of the in-plane component (1) and the out-of-plane component (3), and is characterized by a conical magnetic structure with the cone axis parallel to *c* (Fig. 4d). This type of structure is referred to as conical as the magnetic moments appear to rotate in a conical fashion. Due to the FM contribution to Fmi, all magnetic moments lie parallel to *c* which results in an overall non-zero net moment. Additionally, there is the non-zero contribution of the AFfs with an antiferromagnetic arrangement in the basal plane. Similar to AFfs, the magnetic moments of Fmi are rotated by an angle *φ* from layer to layer.
5. The ferromagnetic mixed commensurate state (Fmc) is a superposition of (2) and (3)—the resulting structure is similar to AFl with the same antiferromagnetic in-plane arrangement and antiferromagnetic coupling between neighboring layers, but the magnetic moments are canted out-of-plane. Thus, Fmc exhibits an additional ferromagnetic coupling along *c* (Fig. 4e).

It should be noted that the FM component (3) is only observed in combination with AFfs (1) and AFl (2) in the Fmi and Fmc structures, and thus, is not an independent magnetic structure of LaMn_2_(Ge_1−*x*_Si_*x*_)_2_. The superposition of magnetic components can be understood as an addition of vectors. Adding an out-of-plane and an in-plane magnetic component will result in a canted magnetic structure. The canting angle of such a non-collinear structure is defined by the ratio of the vector lengths. The individual components are therefore projections onto either the *ab*-plane (AFfs and AFl) or the *c*-axis (FM). Figure 4 illustrates the spin arrangements for all three diffraction conditions and the two observed superpositions of the elementary magnetic contributions.

#### LaMn_2_Ge_2_

**Figure 5:**
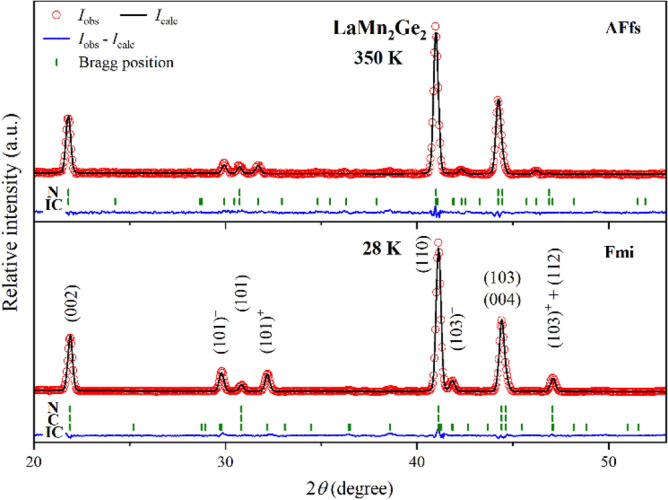
PND patterns of LaMn_2_Ge_2_ at 28 K (*bottom*) and 350 K (*top*). The Bragg markers indicate the positions of the nuclear (*N*), commensurate (*C*) and incommensurate (*IC*) magnetic reflections.

Neutron diffraction data of the ternary LaMn_2_Ge_2_ were collected between 28 and 500 K. Refinements confirm that LaMn_2_Ge_2_ is paramagnetic at 430 K. Below 420 K, magnetic satellite peaks consistent with diffraction condition (1) occur around the (101) and (103) reflections (Fig. 5). They can be indexed with the propagation vector (0, 0, *k*_z_), and their intensities as well as *k*_z_ increase with decreasing temperatures. The magnetic structure is a pure antiferromagnetic flat spiral (AFfs)^28^ (Fig. 4a). The ordering temperature observed here is in good agreement with previous studies^28,34^. At 330 K, slightly above a ferromagnetic-like transition observed in the magnetic susceptibility, the nuclear peaks following reflection condition (3) gain intensity. This increase is most clearly visible on the (112) reflection, as its nuclear contribution is negligible. Diffraction condition (3) describes the ferromagnetic contribution (FM), in which the moments align parallel to *c* (Fig. 4c). The magnetic signal attributed to FM co-exists with the satellite peaks (101)^−^/(101)^+^ and (103)^−^/(103)^+^ of AFfs down to low temperatures. As discussed above, the superposition of an in-plane AFfs and an out-of-plane FM contribution forms the ferromagnetic mixed incommensurate structure (Fmi, Fig. 4d), reported previously^15^.

**Figure 6:**
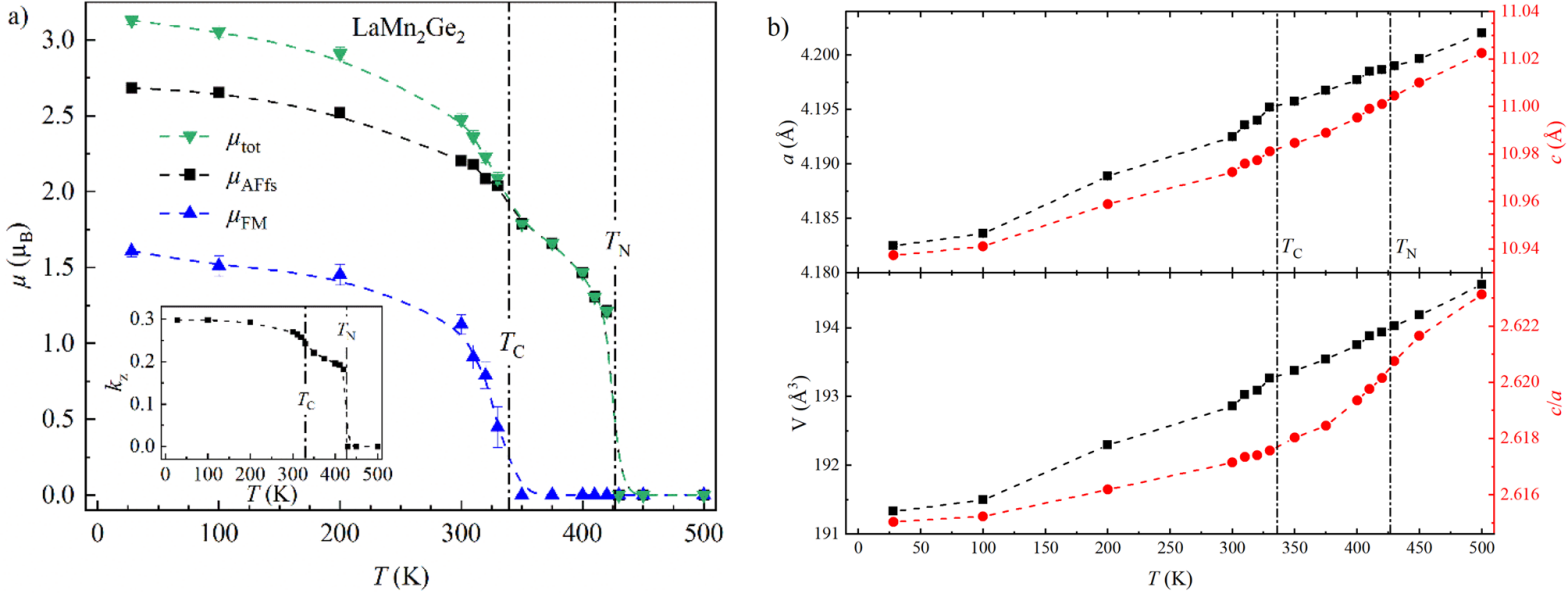
Magnetic and structural parameters of LaMn_2_Ge_2_ derived from PND data refinements: (**a**) Temperature dependence of the total magnetic moment *µ*_tot_, its partial components *µ*_AFfs_, *µ*_FM_, and the propagation vector *k*_z_ (*inset*). (**b**) Change of the lattice parameters *a* and *c*, the unit cell volume *V* and the cell ratio *c*/*a* as a function of temperature. All error bars are shown and represent 1σ. However, the error bars may be smaller than the symbol. The dashed lines connecting neighboring points were added to guide the eye. The vertical dash-dotted lines indicate the magnetic transition temperatures.

Figure 6a shows the temperature dependence of the total magnetic moment *µ*_tot_ of LaMn_2_Ge_2_ and its partial components *µ*_AFfs_ and *µ*_FM_ derived from the data refinements. The magnetic transition temperatures from PND were defined where an abrupt drop in the magnetic moment is observed, as can be seen in Fig. 6 and is indicated by the vertical dash-dotted line. The same methodology was applied to all samples. As it is an approximate value, error propagation is not considered. At 28 K, LaMn_2_Ge_2_ reaches magnetic moments of *µ*_tot_ ≈ 3.13(3) µ_B_, *µ*_AFfs_ ≈ 2.68(2) µ_B_ and *µ*_FM_ ≈ 1.61(4) µ_B_ per Mn with a propagation vector of *k*_z_ ≈ 0.2983(2). At this temperature, the magnetic moment is canted from the *c*-axis by an angle of α ≈ 59.1(4)°. The value of *µ*_FM_ refined from the PND data is slightly larger than the *M*_sat_ value of 1.3005(1) observed in the isothermal magnetization but is in line with the approximately 1.5 µ_B_/Mn reported in the literature^15,40,43–45^. The lower value of *M*_sat_ determined here might be explained by a non-magnetic amorphous impurity or hindered domain wall motion preventing complete saturation of the magnetization. *µ*_tot_ decreases for increasing temperatures, makes a stronger downturn close to *T*_C_ before it vanishes abruptly at 430 K. The intermediate dip at *T*_C_ also occurs in *µ*_AFfs_ and *k*_z_ (Fig. 6a inset). Figure 6b depicts the evolution of the lattice parameters *a* and *c*, the unit cell volume *V* and the cell ratio *c*/*a* as a function of temperature. The cell ratio *c*/*a* exhibits a stronger temperature dependence in the region *T*_C_ < *T* < *T*_N_. This anomalous behavior hints at a strong coupling of the thermal expansion of the crystal lattice to the Mn–Mn inter-layer interactions of the Mn moments. Similar effects have also been observed in CeMn_2_(Ge_1−*x*_Si_*x*_)_2_^11^, PrMn_2_(Ge_1−*x*_Si_*x*_)_2_^31^ and Pr(Mn_1−*x*_Fe_*x*_)_2_Ge_2_^32^.

#### LaMn_2_(Ge_0.95_Si_0.05_)_2_

**Figure 7:**
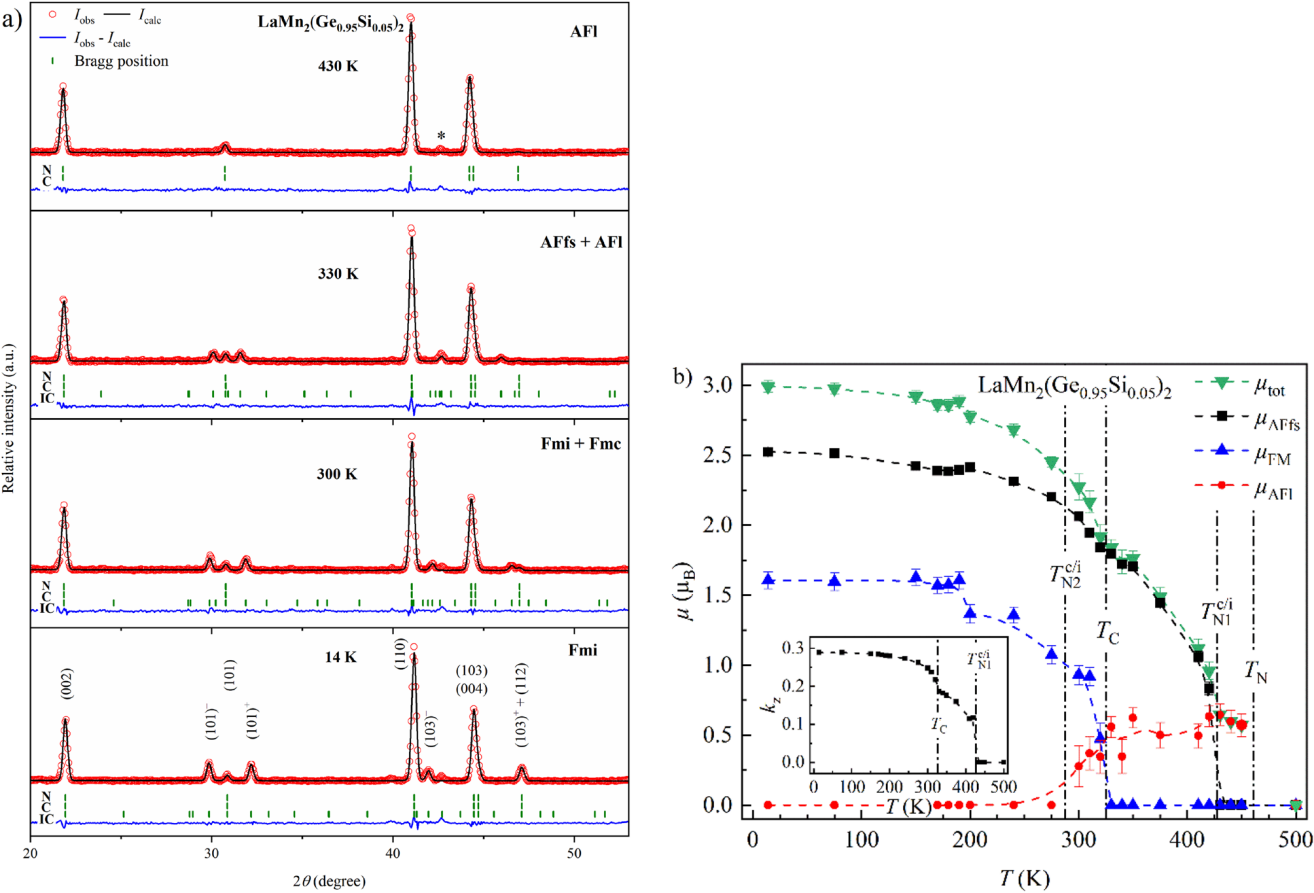
(**a**) PND patterns of LaMn_2_(Ge_0.95_Si_0.05_)_2_ at 14 K, 300 K, 330 K and 430 K (from *bottom* to *top*). The Bragg markers indicate the positions of the nuclear (*N*), commensurate (*C*) and incommensurate magnetic (*IC*) reflections. The asterisk (*) highlights the position of a peak of the impurity La_9.3_((Si_1−*x*_Ge_*x*_)O_4_)_6_O_2_^47^. (**b**) Temperature dependence of the total magnetic moment *µ*_tot_, its partial components *µ*_AFfs_, *µ*_FM_, *µ*_AFl_, and the propagation vector *k*_z_ (*inset*) derived from the PND refinements. All error bars are shown and represent 1σ. However, the error bars may be smaller than the symbol. The dashed lines connecting neighboring points were added to guide the eye. The vertical dash-dotted lines indicate the magnetic transition temperatures.

The introduction of marginal amounts of Si already leads to a significant change in the magnetic properties (Fig. 7a). LaMn_2_(Ge_0.95_Si_0.05_)_2_ is paramagnetic at 500 K. At 450 K, an increased intensity of the (101) reflection, consistent with diffraction condition (2), can be observed, indicating that LaMn_2_(Ge_0.95_Si_0.05_)_2_ orders in the AFl structure. Additional magnetic modulation peaks (101)^−^/(101)^+^ appear at ${T}_{N1}^{c/i}$ ≈ 420 K, signaling the emergence of the AFfs structure, while the magnetic contribution to the (101) reflection does not disappear. That points to a co-existence of the AFl and AFfs phases below 420 K. Thus, ${T}_{N1}^{c/i}$ indicates the transition temperature from a purely AFl component to the co-existing AFfs and AFl and will be used throughout the text. The magnetic moment of the AFl component is significantly smaller than of the AFfs and, therefore, contributes only little to *µ*_tot_ of LaMn_2_(Ge_0.95_Si_0.05_)_2_. The prevalence of the AFfs phase, detected in the ternary LaMn_2_Ge_2_ in a similar temperature region, is in line with the small amount of Si in the solid solution. Below *T*_C_ ≈ 320 K, three magnetic scattering components fulfilling the conditions (1)–(3) co-exist down to ${T}_{N2}^{c/i}$ ≈ 300 K. In line with the terminology used above, ${T}_{N2}^{c/i}$ marks the transition from the co-existing AFfs and AFl contributions to the pure AFfs. The addition of the FM component in this temperature range corresponds to transformation of the AFl and AFfs phases into Fmc and Fmi, respectively. The co-existence of Fmi and Fmc phases was previously reported for the ternary LaMn_2_Si_2_^15,35^. In contrast to related solid solutions with a co-existence of the Fmc structure and the antiferromagnetic mixed commensurate phase (AFmc)^11,29,31,33,46^, a co-refinement of the Fmi and Fmc structures could not be performed for LaMn_2_(Ge_0.95_Si_0.05_)_2_. Since both these magnetic phases share the FM contribution, FM could not be unambiguously partitioned between Fmi and Fmc. Instead, the elementary AFfs, AFl, and FM components were refined individually.

Below 300 K, the AFl contribution vanishes, and only the Fmi magnetic structure remains. The Mn magnetic moment obtained from the refinements at 14 K drops with respect to LaMn_2_Ge_2_ down to *µ*_tot_ ≈ 2.99(4) µ_B_ and *µ*_AFfs_ ≈ 2.52(3) µ_B_. Similarly, the refined value of *k*_z_ ≈ 0.2907(3) is marginally smaller than the value observed in LaMn_2_Ge_2_. The ferromagnetic moment, on the other hand, stays relatively constant at *µ*_FM_ ≈ 1.61(6) µ_B_, which leads to a slightly smaller angle α ≈ 57.5(6)°. The temperature dependence of *µ*_tot_, *µ*_AFfs_ and *k*_z_ shows a strong resemblance to the ternary LaMn_2_Ge_2_ (Fig. 7b). In the temperature region of the co-existing AFl and AFfs phases, *µ*_tot_ was numerically calculated from *µ*_AFl_ and *µ*_AFfs_ and the refinement errors were estimated using the error propagation formula. The in-plane moment of the co-existing *µ*_AFl_ and *µ*_AFfs_ were calculated by averaging over the vector sum using the integral:

1 $${\overline{\mu }}_{AFl+AFfs}=\frac{1}{\pi }{\int }_{0}^{\pi }\sqrt{{\mu }_{AFl}^{2}+{\mu }_{\mathrm{AFfs}}^{2}+2{\mu }_{\mathit{AFl}}{\mu }_{\mathrm{AFfs}}\mathrm{cos\omega }}d\upomega $$

The integral averages over all possible angles *ω* between *µ*_AFl_ and *µ*_AFfs_. For the co-existing Fmi and Fmc phases, the in-plane component calculated using the integral above was combined with the ferromagnetic out-of-plane *µ*_FM_ component using the Pythagorean equation.

#### LaMn_2_(Ge_1−x_Si_x_)_2_ (x = 0.18, 0.33, 0.47, 0.58)

**Figure 8:**
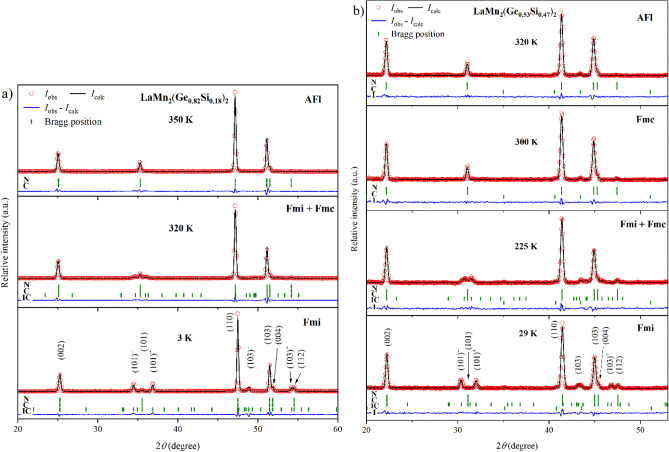
PND patterns of (**a**) LaMn_2_(Ge_0.82_Si_0.18_)_2_ at 3 K, 320 K and 350 K and (**b**) LaMn_2_(Ge_0.53_Si_0.47_)_2_ at 29 K, 225 K, 300 K and 320 K (from *bottom* to *top*). The Bragg markers indicate the positions of the nuclear (*N*), commensurate (*C*) and incommensurate (*IC*) magnetic reflections. LaMn_2_(Ge_0.53_Si_0.47_)_2_ also contains the minor impurity Mn_5_(Ge_1–*x*_Si_*x*_)_3_ (*I*).

A further increase in the Si fraction in LaMn_2_(Ge_1−*x*_Si_*x*_)_2_ leads to a continuous increase of *T*_N_ and the disappearance of the AFfs structure. In the samples with compositions LaMn_2_(Ge_1−*x*_Si_*x*_)_2_ (*x* = 0.18, 0.33, 0.47, 0.58), only AFl could be observed above *T*_C_ (Fig. 8). LaMn_2_(Ge_0.67_Si_0.33_)_2_ retains this magnetic structure up to at least 475 K. In LaMn_2_(Ge_0.53_Si_0.47_)_2_, the paramagnetic regime was not reached even at 500 K. For the other two samples (*x* = 0.18, 0.58) no data were collected at such high temperatures. The composition-dependent increase of the Néel temperature follows the trend previously detected by Perturbed Angular Correlation (PAC) spectroscopy^34^.

In LaMn_2_(Ge_0.82_Si_0.18_)_2_, the simultaneous appearance of the satellite peaks (101)^−^/(101)^+^ and the intensity increase of the (112) reflection below 320 K indicate a transition to the Fmi structure below *T*_C_ (Fig. 8a). However, as the magnetic scattering contribution on the (101) reflection does not disappear at the same time, the Fmi co-exists with the Fmc phase down to at least 290 K. Finally, LaMn_2_(Ge_0.82_Si_0.18_)_2_ transforms into the Fmi structure at even lower temperatures. Since only a few data points were collected for this sample, the exact transition temperature is not known but it occurs somewhere between 200 K < ${T}_{N2}^{c/i}$  < 290 K. Taking the transition temperatures of the neighboring samples into account, ${T}_{N2}^{c/i}$ is expected to be at around 250 K.

Below *T*_C_, the samples with the compositions *x* = 0.33, 0.47, 0.58 undergo a transition to the Fmc phase, identified by magnetic peaks consistent with diffraction conditions (2) and (3). Below 275 K, 250 K and 210 K, respectively, modulation peaks following condition (1) appear, which suggests co-existence of the Fmc and Fmi structures. Interestingly, the temperature range in which this co-existence is observed increases with the amount of Si in LaMn_2_(Ge_1−*x*_Si_*x*_)_2_: at 275 K < *T* < 250 K for *x* = 0.33, at 250 K < *T* < 200 K for *x* = 0.47, and at 210 K < *T* < 70 K for *x* = 0.58. Figure 8b shows the PND patterns of LaMn_2_(Ge_0.53_Si_0.47_)_2_ at 320 K, 300 K, 225 K and 29 K.

**Figure 9:**
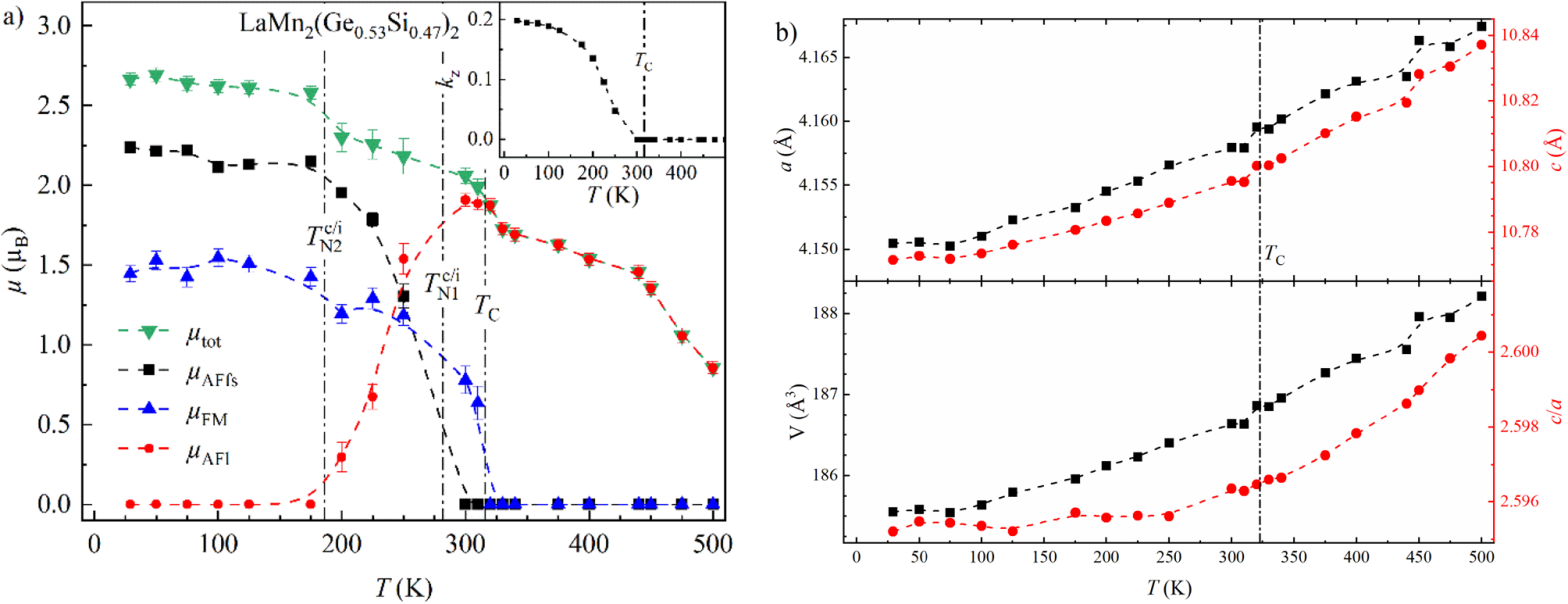
Magnetic and structural parameters of LaMn_2_(Ge_0.53_Si_0.47_)_2_ derived from the refinements of the PND data: (**a**) Temperature dependence of the total magnetic moment *µ*_tot_, its partial components *µ*_AFfs_, *µ*_FM_, and the propagation vector *k*_z_ (*inset*). (**b**) Change of the lattice parameters *a* and *c*, the unit cell volume *V* and the cell ratio *c*/*a* as a function of temperature. All error bars are shown and represent 1σ. However, the error bars may be smaller than the symbol. The dashed lines connecting neighboring points were added to guide the eye. The vertical dash-dotted lines indicate the magnetic transition temperatures.

The total magnetic moment of LaMn_2_(Ge_0.53_Si_0.47_)_2_ reaches *µ*_tot_ ≈ 2.66(4) µ_B_ per Mn atom, with partial components of *µ*_AFfs_ ≈ 2.24(3) µ_B_ and *µ*_FM_ ≈ 1.44(5) µ_B_ at 29 K and a resulting angle of α ≈ 59.2(6)°. Thus, both magnetic contributions drop compared to the Ge-richer samples. In a similar fashion, a lower *k*_z_ value of 0.1983(3) is observed. The temperature dependence of *µ*_tot_, the partial magnetic moments *µ*_AFfs_, *µ*_AFl_, *µ*_FM_, and *k*_z_ are plotted in Fig. 9a. It is noteworthy that *µ*_AFl_ rises while *µ*_AFfs_ drops in the temperature region where Fmi and Fmc co-exist. Meanwhile, *µ*_FM_ appears to be unperturbed. Thus, the co-existence occurs in the temperature region where the phase transition from Fmc to Fmi takes place. In comparison to LaMn_2_Ge_2_ and LaMn_2_(Ge_0.95_Si_0.05_)_2_, *k*_z_ decreases less abruptly in LaMn_2_(Ge_0.53_Si_0.47_)_2_ (Fig. 9a inset). Figure 9b shows the change of the lattice parameters as a function of temperature. The non-linear behavior of *c*/*a* with a slope increase around *T*_C_ noted earlier for LaMn_2_Ge_2_ can also be seen in LaMn_2_(Ge_0.53_Si_0.47_)_2_ but becomes less pronounced with increasing Si-concentration.

#### LaMn_2_(Ge_1−x_Si_x_)_2_ (x = 0.78, 1)

PND patterns of the samples LaMn_2_(Ge_0.22_Si_0.78_)_2_ and LaMn_2_Si_2_ were collected between 3 and 295 K. At room temperature, both adopt the Fmc structure, identified by magnetic peaks following conditions (2) and (3). LaMn_2_(Ge_0.22_Si_0.78_)_2_ preserves this structure down to 150 K. At lower temperatures, the (101)^−^/(101)^+^ reflections of the incommensurate AFfs emerge, in addition to the magnetic peaks satisfying conditions (2) and (3). Thus, the co-existence of Fmi and Fmc structure is also observed in LaMn_2_(Ge_0.22_Si_0.78_)_2_ and retained down to 3 K.

**Figure 10:**
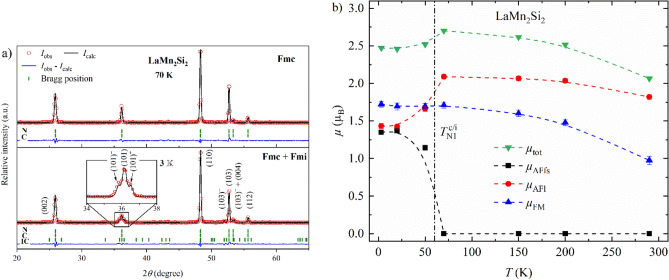
(**a**) Powder neutron diffraction patterns of LaMn_2_Si_2_ at 3 K (*bottom*) and 70 K (*top*). The Bragg markers indicate the positions of the nuclear (*N*), commensurate (*C*) and incommensurate magnetic (*IC*) reflections (from *top* to *bottom*). (**b**) Temperature dependence of the total magnetic moment *µ*_tot_ and its partial components *µ*_AFfs_, *µ*_AFl_ and *µ*_FM_. All error bars are shown and represent 1σ. However, the error bars may be smaller than the symbol. The dashed lines connecting neighboring points were added to guide the eye. The vertical dash-dotted line indicate the magnetic transition temperatures.

Interestingly, LaMn_2_Si_2_ orders in the same magnetic structures as LaMn_2_(Ge_0.22_Si_0.78_)_2_: LaMn_2_Si_2_ remains in the Fmc phase down to 70 K, and the co-existence of Fmi and Fmc sets in at 50 K (Fig. 10a). In previous PND measurements of LaMn_2_Si_2_, the satellite reflections (101)^−^/(101)^+^ were detected as a broadening at the foot of the (101) peak^15,35^. We can clearly distinguish the satellite peaks thanks to the higher resolution of our data. The incommensurate peaks were refined with *k*_z_ ≈ 0.0710(5) at 3 K which is even smaller than the value of 0.09 reported earlier^15,35^. The temperature dependence of the total magnetic moment *µ*_tot_ and its partial components *µ*_AFfs_, *µ*_AFl_ and *µ*_FM_ is shown in Fig. 10b. The magnetic moments vary substantially from previous studies. The value for *µ*_AFfs_ derived from our refinements is 1.35(3) µ_B_ at 3 K, which is significantly larger than the 0.8 µ_B_ and 0.5 µ_B_ published by Venturini et al.^15^ and Hofmann et al., respectively^35^. *µ*_FM_ ≈ 1.72(4) µ_B_ is in line with their results but *µ*_AFl_ ≈ 1.43(3) µ_B_ is slightly lower. Nevertheless, *µ*_tot_ reaches similar values in all cases. We attribute the discrepancies between the partial moments to our improved data resolution. As we were able to resolve the modulation peaks, it is much easier to refine the accurate values of *k*_z_ and the partial magnetic moments.

**Figure 11:**
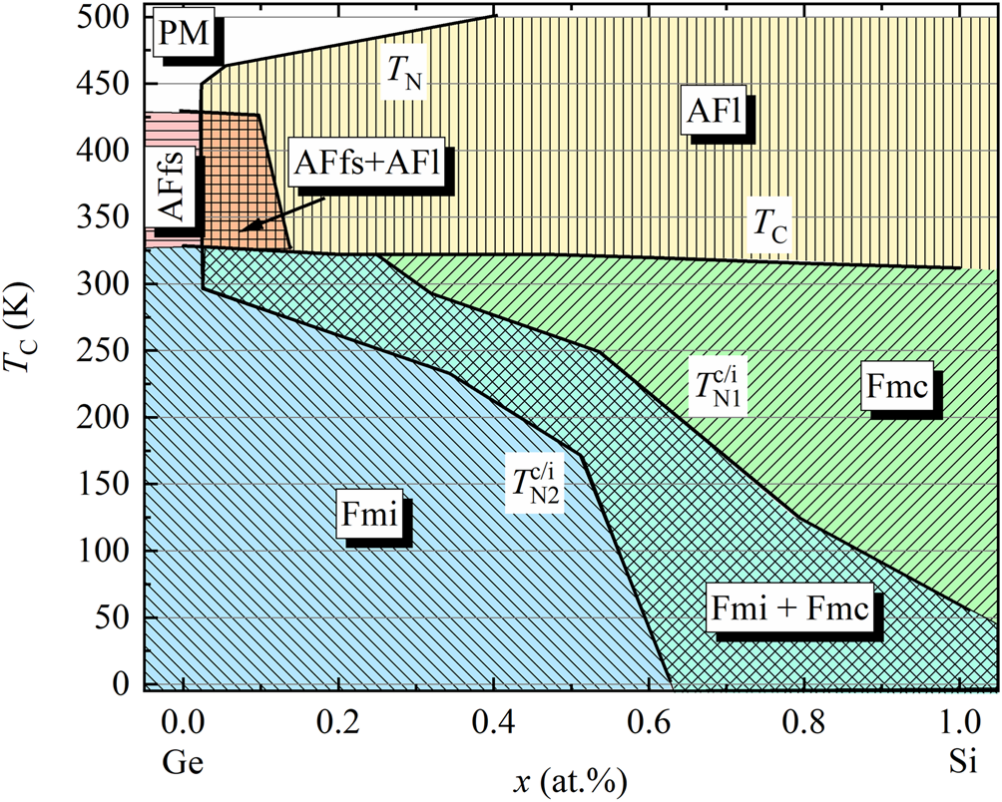
*x*-*T* magnetic phase diagram of the solid solution LaMn_2_(Ge_1-*x*_Si_*x*_)_2_: Paramagnetic (PM), antiferromagnetic flat spiral (AFfs), antiferromagnetic layers (AFl), ferromagnetic mixed incommensurate (Fmi), ferromagnetic mixed commensurate (Fmc). The magnetic phase boarders were determined from the PND data. The slight composition-dependent shift of *T*_C_ was established from the magnetic susceptibility.

The compositional and thermal variation of the magnetic phases is plotted in Fig. 11. Below *T*_C_, the Ge-rich part of the solid solution is dominated by the Fmi phase, the Si-rich—by Fmc. In-between, the co-existence of Fmi and Fmc originally observed in LaMn_2_Si_2_^15,35^ spreads from low Si concentrations at high temperatures to the Si-rich compositions at low temperatures. Fmi and Fmc co-exist in every quaternary sample as well as in LaMn_2_Si_2_. As the FM component vanishes at *T*_C_, the magnetic moments align within the plane of the Mn square net. The AFl phase prevails for a wide range of compositions at *T*_C_ < *T* < *T*_N_, while AFfs is favored by LaMn_2_Ge_2_. In the narrow Si-poor window around *x* = 0.05, AFfs and AFl co-exist above *T*_C_. As the AFfs contribution vanishes faster than the AFl, the pure AFl structure is detected at higher temperatures. The thick black lines in Fig. 11 sketch the phase edges. The composition dependence of *T*_C_ was plotted using the values in Table 1. Supplementary Fig. S6 presents another version of the same magnetic *x*-*T* phase diagram where all measured temperature points are displayed.

## Discussion

Co-existence of different magnetic phases is frequently observed in solid solutions. In the ThCr_2_Si_2_-type structure alone it has been found for, e.g., La_1−*x*_Y_*x*_Mn_2_Si_2_^29,46,48^_,_ La_1−*x*_Pr_*x*_Mn_2_Si_2_^33^, CeMn_2_(Ge_1−*x*_Si_*x*_)_2_^11^ and PrMn_2_(Ge_1−*x*_Si_*x*_)_2_^31^. In all these examples, such co-existence is reported in a limited composition region. LaMn_2_(Ge_1−*x*_Si_*x*_)_2_ sets itself apart from all these cases as the co-existence occurs in all quaternary samples *and* the ternary LaMn_2_Si_2_. In the literature, the origin of magnetic phase co-existence is usually explained by a chemical phase separation of the quaternary samples into regions with nearly identical compositions and, thus, nearly identical lattice parameters. In CeMn_2_(Ge_1−*x*_Si_*x*_)_2_, for example, compositional inhomogeneity was suggested based on high-resolution synchrotron PXRD studies^11^. In La_1−*x*_Y_*x*_Mn_2_Si_2_, a peak splitting could even be observed in the PND data^48^. The coexistence of two or more magnetic phases found in the PND measurements is supported by the non-saturation of the isothermal magnetization found for LaMn_2_Si_2_ and some of the quaternary samples at 2 K under 6 T external field. This behavior of the isothermal magnetization indicates the existence of more than one magnetic component, as explained above.

**Figure 12:**
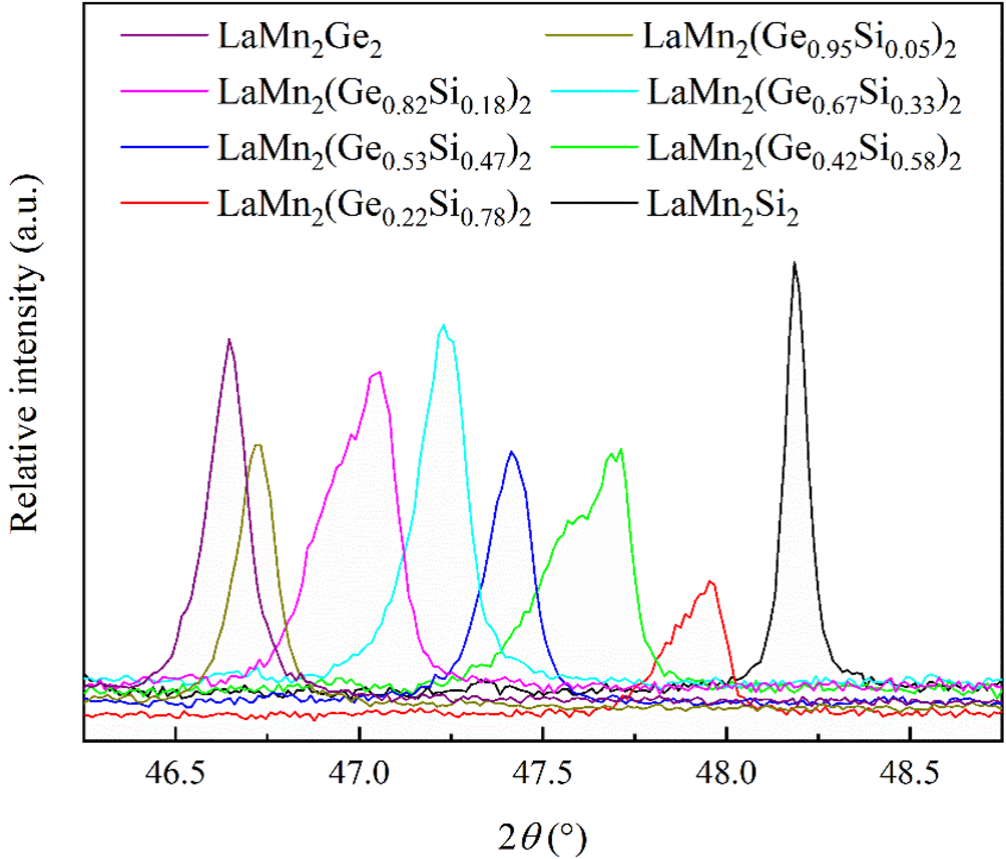
PXRD patterns of LaMn_2_(Ge_1-*x*_Si_*x*_)_2_ at room temperature in the 2*θ* region around the (105) reflection.

In the quaternary samples in our study, a broadening of certain peaks in the PXRD data at room temperature can be detected, which likely indicates a somewhat inhomogeneous distribution of Si and Ge. Since this behavior is especially visible for some (*hkl*) reflections with non-zero *l*, these small inhomogeneities must have a stronger impact on *c*. Figure 12 shows the PXRD patterns in the 2*θ* region around the (105) reflection. The peak broadening and the asymmetry is pronounced in some of the quaternary samples. In LaMn_2_(Ge_0.82_Si_0.18_)_2_ and LaMn_2_(Ge_0.42_Si_0.58_)_2_, the (105) reflection even appears to be split. LaMn_2_Si_2_, however, exhibits the smallest reflection width, rendering any significant chemical inhomogeneity (such as related to intrinsic defects) improbable. Future high-resolution PXRD measurements at a synchrotron source may shed light on the crystal structural origin of the co-existing magnetic phases in LaMn_2_Si_2_.

The partial substitution of Ge by Si leads to a minor decrease of *T*_C_ from 326.10(4) K in LaMn_2_Ge_2_ to 308.37(6) K in LaMn_2_Si_2_ and was reported previously^36^. The values for *T*_C_ we observe from magnetization and PND measurements are in agreement with each other and match those from the literature^28,36,49^. Considering the strong composition and temperature dependence of the AFfs and AFl components (Fig. 11 and Supplementary Fig. S6) it is noteworthy that FM, and therefore *T*_C_, remains nearly constant throughout the solid solution. A similar effect was also noted for *T*_N_, which increases monotonically with increasing Si content from approximately 420 K in LaMn_2_Ge_2_^34^ to 470 K in LaMn_2_Si_2_ according to the literature^34,35,49,50^. The Neél temperature of LaMn_2_Ge_2_ is in line with the values reported earlier^28,34^. Although we did not investigate the high temperature behavior for all samples, the three quaternary samples (*x* = 0.05, 0.33, 0.47) for which we collected PND data up to 500 K confirm the trend observed earlier: *T*_N_ increases with increasing Si content^34^. Our data suggests, however, that the actual ordering temperatures may be higher than reported previously^34,35,50^. This is especially visible for the sample with the composition *x* = 0.47 which did not even reach the paramagnetic regime up to 500 K. Additional measurements at elevated temperatures may be required to confirm if *T*_N_ is indeed higher than the values reported in the literature.

**Figure 13:**
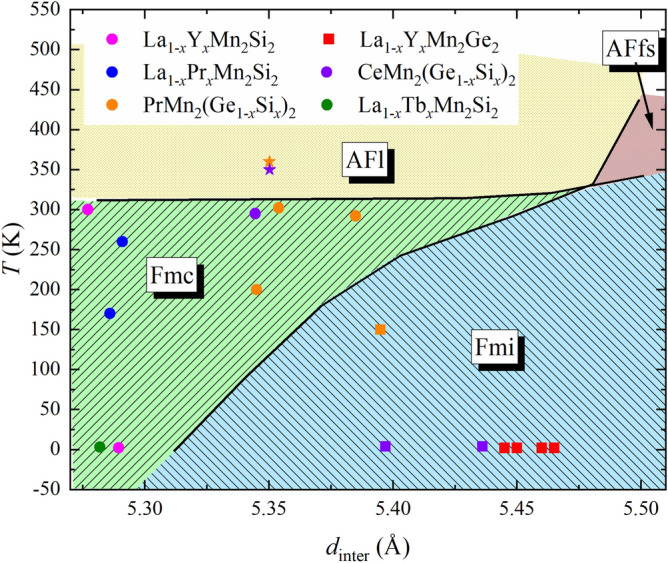
Universal magnetic *d*_inter_-*T* phase diagram of the *RE*Mn_2_*X*_2_ systems. Colored regions are based on the data of the solid solution LaMn_2_(Ge_1-*x*_Si_*x*_)_2_. Additional data points for Fmc (filled circle), Fmi (filled square) and AFl (filled star) from other solid solutions were added for comparison^11,28,29,31,33,38^.

The *x*-*T* phase diagram of LaMn_2_(Ge_1−*x*_Si_*x*_)_2_ exhibits certain similarities to those of La_1−*x*_Y_*x*_Mn_2_Ge_2_^28^, CeMn_2_(Ge_1−*x*_Si_*x*_)_2_^11^ and PrMn_2_(Ge_1−*x*_Si_*x*_)_2_^31^. In all these solid solutions, the Fmc structure is observed in a similar composition range as Fmi. Analysis of the unit cell and magnetic phase evolution indicates that the Fmi structure dominates the samples with longer lattice parameters and at lower temperatures, while Fmc is found for the samples with shorter lattice parameters and at higher temperatures^11,28,31^. The same tendency is observed in LaMn_2_(Ge_1−*x*_Si_*x*_)_2_ and suggests a correlation to the lattice dimensions. In previous studies, the intra-planar Mn–Mn distance was proposed as one of the important crystal structure parameters that help rationalize the magnetic phase diagram of the *RE*Mn_2_*X*_2_ systems, as was discussed in the Introduction. We note, however, that the distance between adjacent Mn square nets also appears to be a significant factor for stabilization of certain magnetic phases. As the composition dependence of *c* does not follow Vegard’s law but appears to make a kink in Ge-rich side of the solid solution (Fig. 2), the composition region where magnetic incommensurability is most pronounced even above room temperature (Fig. 11), the transition from incommensurate to commensurate structure must be governed by the Mn–Mn interlayer spacing *d*_inter_. Figure 13 shows the *d*_inter_-*T* phase diagram of the different magnetic structures in LaMn_2_(Ge_1−*x*_Si_*x*_)_2_ which can be assigned to clearly defined regions. The data points of the co-existing phases were excluded for this consideration. Published results for other solid solutions series were added to the phase diagram in order to put the *d*_inter_-*T* trend found from our data into perspective. Interestingly, each data point from these other solid solutions fits perfectly into the *d*_inter_-*T* phase diagram of LaMn_2_(Ge_1−*x*_Si_*x*_)_2_. Thus, the occurrence of the commensurate Fmc and incommensurate Fmi structures can be directly correlated to the inter-planar Mn–Mn distances and the temperature. Therefore, Fig. 13 represents a “universal” phase diagram for the *RE*Mn_2_*X*_2_ systems. Although it may not enable prediction of all possible magnetic phases in these materials, the uncovered relationship between the magnetism and the crystal structure can be used to target magnetic incommensurability, which can be of significance for design of functional magnetic materials.

## Conclusions

The influence of the substitution of Ge by Si in LaMn_2_(Ge_1−*x*_Si_*x*_)_2_ on the structural and magnetic properties has been investigated by PXRD, magnetization and PND measurements between 3 and 500 K, which allowed establishing a magnetic phase diagram. Replacing Ge with Si leads to a compression of the unit cell. The non-linear lattice contraction in the Ge-richer samples at room temperature suggests strong magnetovolume effects.

The magnetic structures of LaMn_2_(Ge_1−*x*_Si_*x*_)_2_ are strongly affected by the change of the unit cell parameter *c*, which is reflective of the interlayer separation. In the *x*-*T* phase diagram, the commensurate Fmc and AFl structures dominate the Si-richer part of the solid solution mostly at higher temperatures, while the incommensurate Fmi and AFfs prevail in the Si-poorer part at lower temperatures. Thus, the transition from commensurate to incommensurate phases is linked to a combination of both inter-planar Mn–Mn distances and temperature. Co-existence of magnetic phases is observed in all quaternary samples and LaMn_2_Si_2_. Peak broadening of certain reflections in the PXRD pattern of the quaternary samples suggests the existence of compositional inhomogeneities as a result of the Ge/Si mixing. This effect could be the origin of the magnetic phase co-existence in the quaternary compositions. However, the same cannot be true for LaMn_2_Si_2_. High-resolution PXRD measurements might shed light on the origin of the co-existence of magnetic phases in LaMn_2_Si_2_. Comparison of the data on the LaMn_2_(Ge_1−*x*_Si_*x*_)_2_ series and related solid solutions reported in the literature allows construction of a universal phase diagram relating the emergence of magnetic incommensurability to the inter-planar Mn–Mn distance.

## Methods

### Synthesis

LaMn_2_(Ge_1−*x*_Si_*x*_)_2_ samples with the composition *x* = 0, 0.05, 0.33, 0.47 were produced at the Department for Materials and Environmental Chemistry at Stockholm University by first preparing the LaGe precursor through arc-melting of a 1:1 stoichiometric mixture of the elements (La 99.99% purity, Ge 99.999%). To synthetize the respective LaMn_2_(Ge_1−*x*_Si_*x*_)_2_ compositions, LaGe was mixed with the appropriate amounts of elemental Mn (99.95%), Si (99.999%), and Ge. The nominal compositions corresponded to *x* = 0, 0.1, 0.4, and 0.5. The mixtures were thoroughly ground, pelletized, and wrapped in Mo foil. The pellets were then enclosed in evacuated fused silica tubes under a pressure of approximately 0.1 Pa and annealed at 1273 K for 2 weeks in a tube furnace with several intermediate regrinding/repelletizing steps. After each annealing step, the samples were allowed to cool to room temperature naturally by shutting-off the furnace.

LaMn_2_(Ge_1−*x*_Si_*x*_)_2_ samples with *x* = 0.18, 0.58, 0.78, 1 were prepared in the Hyperfine Interactions Laboratory at the Instituto de Pesquisas Energéticas e Nucleares (IPEN). Starting elements were molten in an argon atmosphere purified with a hot titanium getter. La pieces with 99.9% of purity and Mn, Ge and Si pieces with 99.999% of purity were added in the stoichiometric ratio. A little excess of Mn (around 5% by mass fraction) was used to compensate the weight loss by evaporation during reaction. After melting, the resulting ingot of each sample was sealed in an evacuated fused silica tube under reduced pressure of 10^–2^ Pa and annealed at 1073 K for 24 h.

The sample composition was confirmed by powder X-ray diffraction (PXRD) and revealed, aside from the targeted pseudo ternary, small amounts of impurities for the samples with the composition *x* = 0, 0.05, 0.33, 0.47 and 0.58, thus resulting in slight deviations of the major phase composition from the nominal one. The following impurities were identified: La_2_O_3_^51^ in *x* = 0 [0.92(5) % by mass fraction]; La_9.3_((Si_1−*x*_Ge_*x*_)O_4_)_6_O_2_^47^ in *x* = 0.05 [0.84(9) % by mass fraction], 0.33 [1.47(8) % by mass fraction] and 0.47 [1.20(9) % by mass fraction]; Mn_5_(Ge_1–*x*_Si_*x*_)_3_^52,53^ in *x* = 0.33 [3.18(13) % by mass fraction] and 0.47 [3.03(14) % by mass fraction]; La_5_(Ge_1–*x*_Si_*x*_)_4_^54^ in *x* = 0.58 [1.49(19) % by mass fraction]. The compositions for the respective LaMn_2_(Ge_1−*x*_Si_*x*_)_2_ samples were refined from the PXRD data and are used throughout the text to identify the samples.

### Powder X-ray diffraction (PXRD)

Powder X-ray diffraction patterns were collected at room temperature using a Panalytical X’Pert PRO diffractometer (Panalytical, Netherlands) operated in Bragg-Brentano geometry. The instrument is equipped with a Johansson Ge monochromator to generate pure Cu *K*_α1_ radiation (*λ* = 1.54059 Å). The samples were measured on zero-background Si sample holders. Rietveld refinements of the PXRD patterns were performed by Fullprof^55^. Phase analysis yielded only small amounts of impurities. Three representative PXRD patterns of the samples with the composition *x* = 0, 0.47 and 1 are plotted in Supplementary Fig. S2a–c.

### Magnetic measurements

Magnetization was measured utilizing a Quantum Design Physical Property Measurement System (PPMS, Quantum Design, USA). A Vibrating Sample Magnetometer (VSM) option was employed to collect zero-field cooled (ZFC) and field-cooled (FC) magnetization data between 2 and 400 K in static magnetic fields (DC). Isothermal magnetization was measured at 2 K and 250 K up to 6 T. Polycrystalline samples were loaded into polypropylene (PP) sample containers which were subsequently mounted in brass sample holders.

### Powder neutron diffraction (PND)

Powder neutron diffraction patterns were acquired during two beamtimes at the neutron sources of the Canadian Neutron Beam Centre (CNBC, Chalk River, Ontario, Canada) and the Center for High Resolution Neutron Scattering (CHRNS) at the National Institute of Standards and Technology (NIST Center for Neutron Research (NCNR), Gaithersburg, MD, USA), respectively. At the CNBC, diffraction patterns for the LaMn_2_(Ge_1−*x*_Si_*x*_)_2_ samples with *x* = 0.18, 0.58, 0.78 and 1 were collected on the High Resolution Powder Diffractometer C2 in the angular range 2*θ* between 18.9° to 99° with a neutron wavelength of *λ* = 2.3722(17) Å in a He-cryostat (3 K to 290 K) and a dedicated furnace (320 K to 380 K). At NCNR, the measurements with the compositions *x* = 0, 0.05, 0.33 and 0.47 took place at the High Resolution Neutron Powder Diffractometer BT-1^56^ equipped with 32 ^3^He detectors covering an angular range of 3° ≤ 2*θ* ≤ 166° with a step size of 0.050°. The data were collected using a Ge (311) monochromator wavelength of *λ* = 2.0787(2) Å and in pile collimation of 60 min per arc. Closed Cycle Refrigerators (CCRs) were used to cover the temperature range of 14 K to 500 K. Rietveld refinements of the PND patterns were carried out for magnetic structure determination employing Fullprof for all samples^55^.

## Supplementary Information

Supplementary Information.

## Data Availability

The datasets generated during and/or analyzed during the current study are available from the corresponding author on reasonable request.
