# Supplementary material for: Magnetic phase diagram of the solid solution LaMn2(Ge1−xSix)2 (0 ≤ x ≤ 1) unraveled by powder neutron diffraction
Source: Sci Rep. 2022 Jun 3;12:9248. doi: 10.1038/s41598-022-12549-y (PMC9166718; doi:10.1038/s41598-022-12549-y)
Supplement: Supplementary file 1 — Supplementary Information. [file 41598_2022_12549_MOESM1_ESM.pdf]

Supporting Information: Magnetic phase diagram of the solid solution $LaMn_2(Ge_{1-x}Si_x)_2$ ($0 \leq x \leq 1$) revealed by powder neutron diffraction

Stefanie Siebeneichler,[a] Alexander Ovchinnikov,[a] Brianna Bosch-Santos,[b, c] Gabriel A. Cabrera-Pasca,[d] Roxana Flacau,[e] Qingzhen Huang,[f] Artur W. Carbonari,[c] Dominic Ryan,[g] and Anja-Verena Mudring*, [a, h]

[a] Department of Materials and Environmental Chemistry, Stockholm University, Svante Arrhenius väg 16 C, 10691 Stockholm, Sweden.

[b] Material Measurement Laboratory, National Institute of Standards and Technology - NIST, Gaithersburg, MD 20899, USA.

[c] Instituto de Pesquisas Energéticas e Nucleares – IPEN-CNEN/SP, São Paulo, SP, 05508-000, Brazil.

[d] Programa de Pós-Graduação em Ciência e Engenharia de Materiais – PPGCEM, Universidade Federal do Pará, Ananindeua, PA, 67130 660, Brazil.

[e] Canadian Neutron Beam Centre, Chalk River Laboratories, Chalk River, Ontario K0J 1J0, Canada.

[f] Center for Neutron Research, National Institute of Standards and Technology, Gaithersburg, MD, 20899, USA.

[g] The Centre for the Physics of Materials and the Physics Department, McGill University, 3600 University St., Montreal (Quebec) H3A 2T8, Canada.

[h] Department of Chemistry, Aarhus University, Langelandsgade 140, 8000 Aarhus C, Denmark.

* Corresponding author: anja-verena.mudring@mmk.su.se; anja-verena.mudring@chem.au.dk

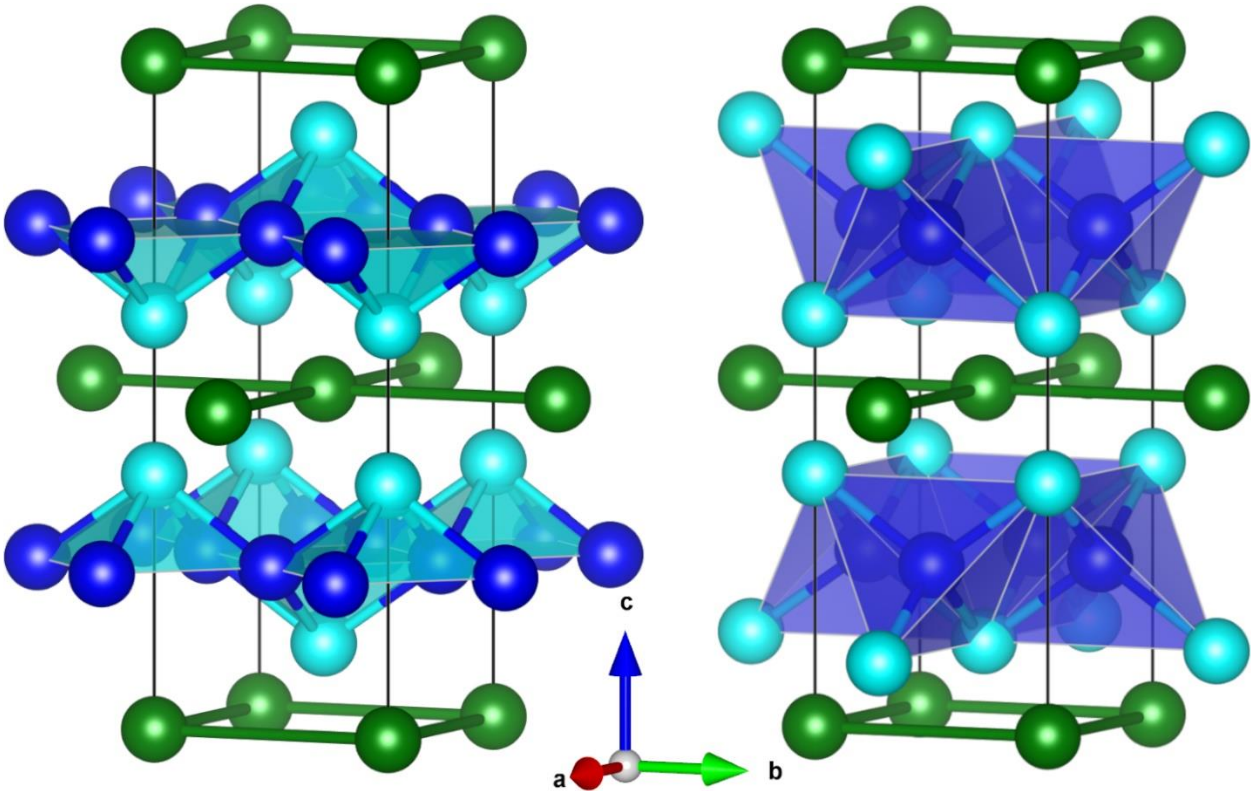

Figure S1: Alternative descriptions of the $LaMn_2(Ge_{1-x}Si_x)_2$ crystal structure illustrating layers of edge-sharing $GeMn_4$ square pyramids with alternating orientation (*left*) and layers of edge-sharing $MnGe_4$ tetrahedra (*right*), respectively, alternating with square nets of La: La (*green*), Mn (*dark blue*), Si/Ge (*turquoise*).

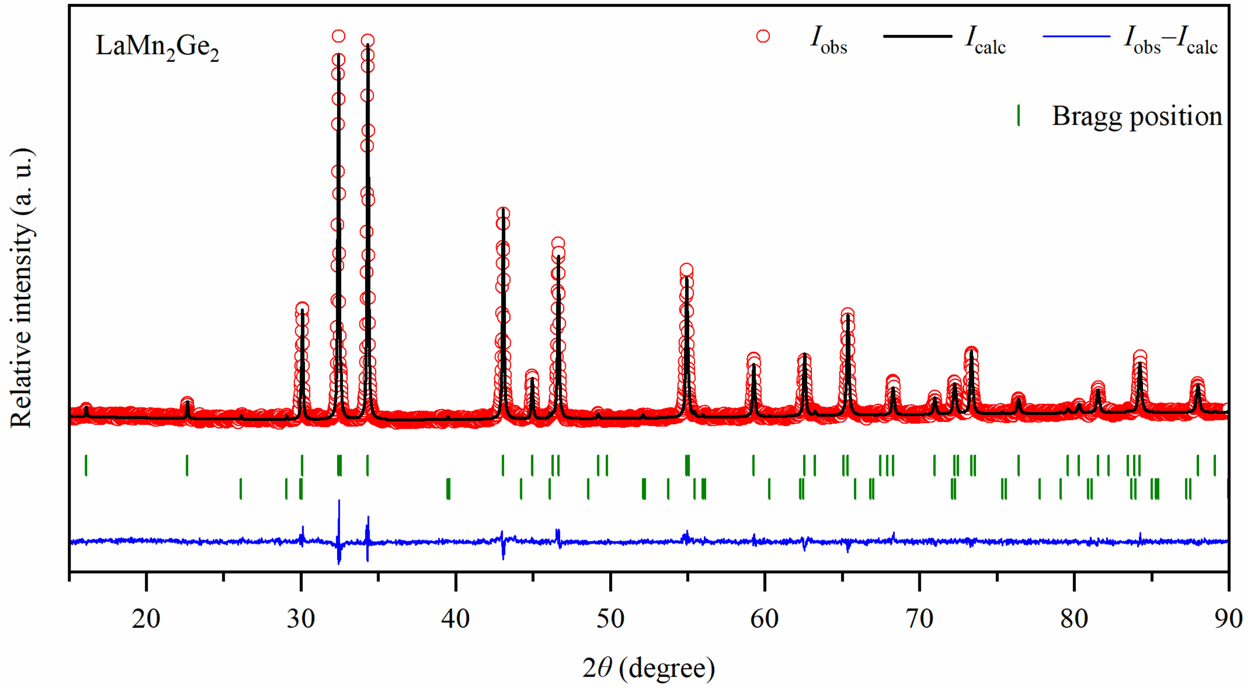

Figure S2a: PXRD pattern of $LaMn_2Ge_2$. The Bragg markers indicate the reflection positions for $LaMn_2Ge_2$ (*top*) and the minor impurity La_2O_3[1] (*bottom*).

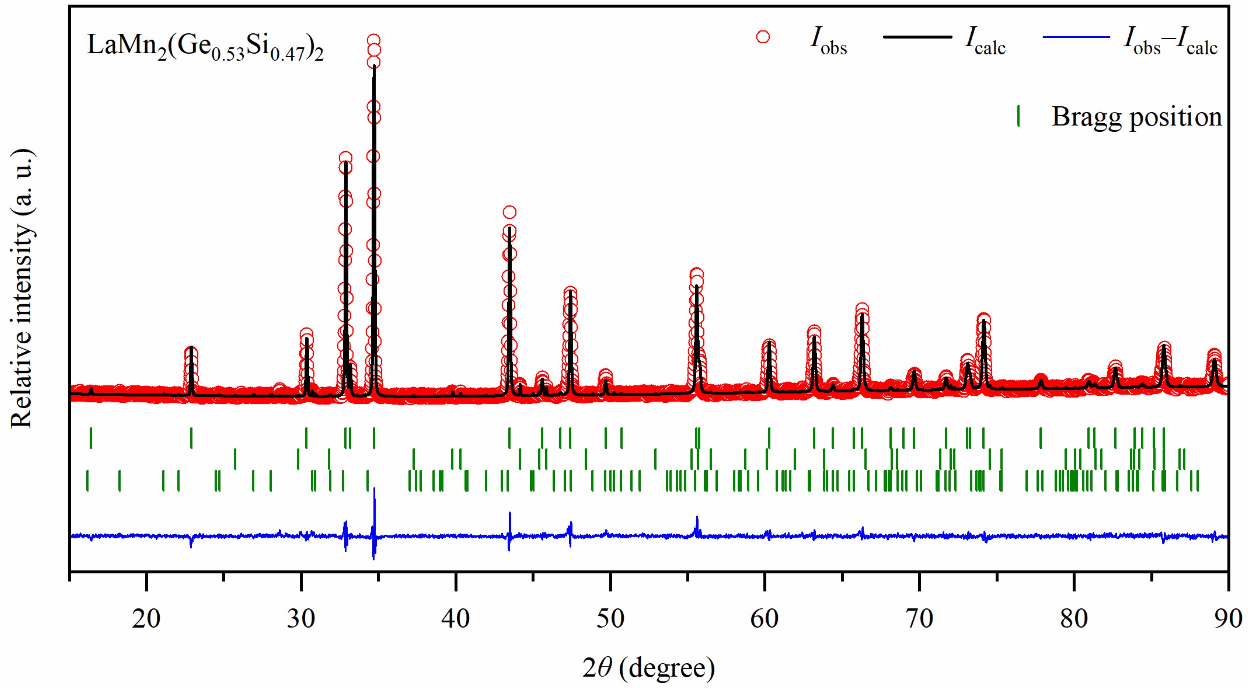

Figure S2b: PXRD pattern of $LaMn_2(Si_{0.47}Ge_{0.53})_2$. The Bragg markers indicate the reflection positions for $LaMn_2(Si_{0.47}Ge_{0.53})_2$ (*top*) and the minor impurities $Mn_5(Si_{1-x}Ge_x)_3$[2] (*middle*) and $La_{9.3}((Si_{1-x}Ge_x)O_4)_6O_2$[3] (*bottom*).

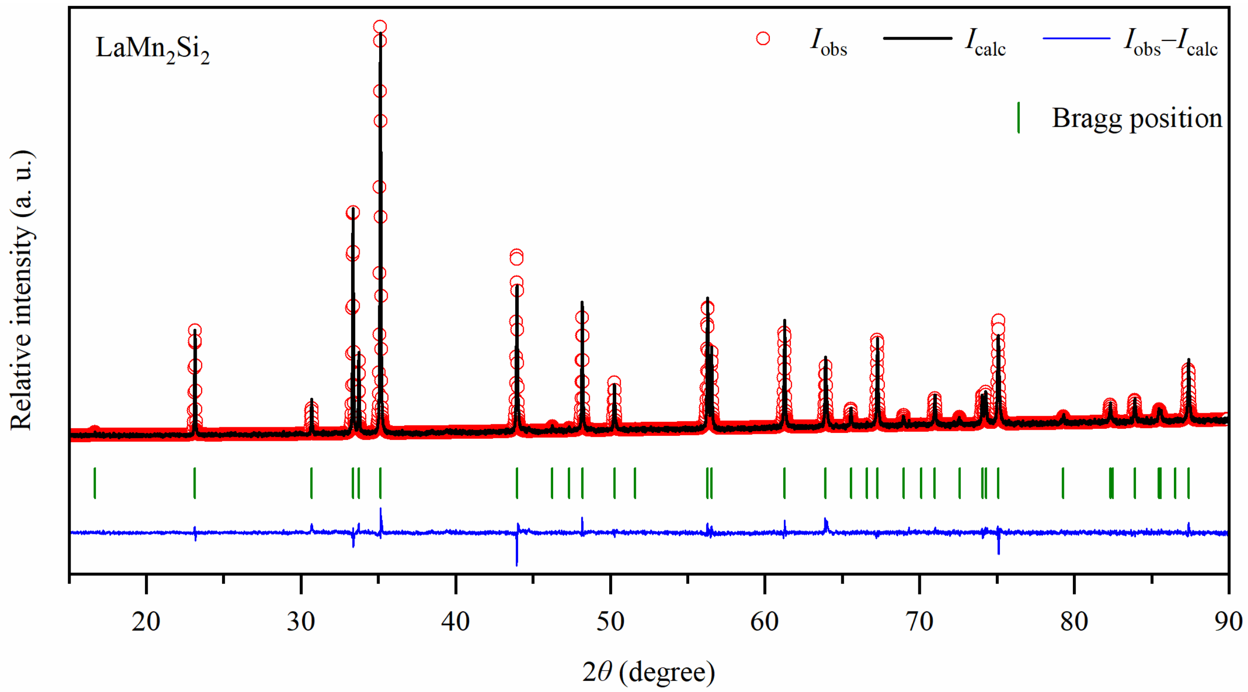

Figure S2c: PXRD pattern of $LaMn_2Si_2$. The Bragg markers indicate the reflection positions.

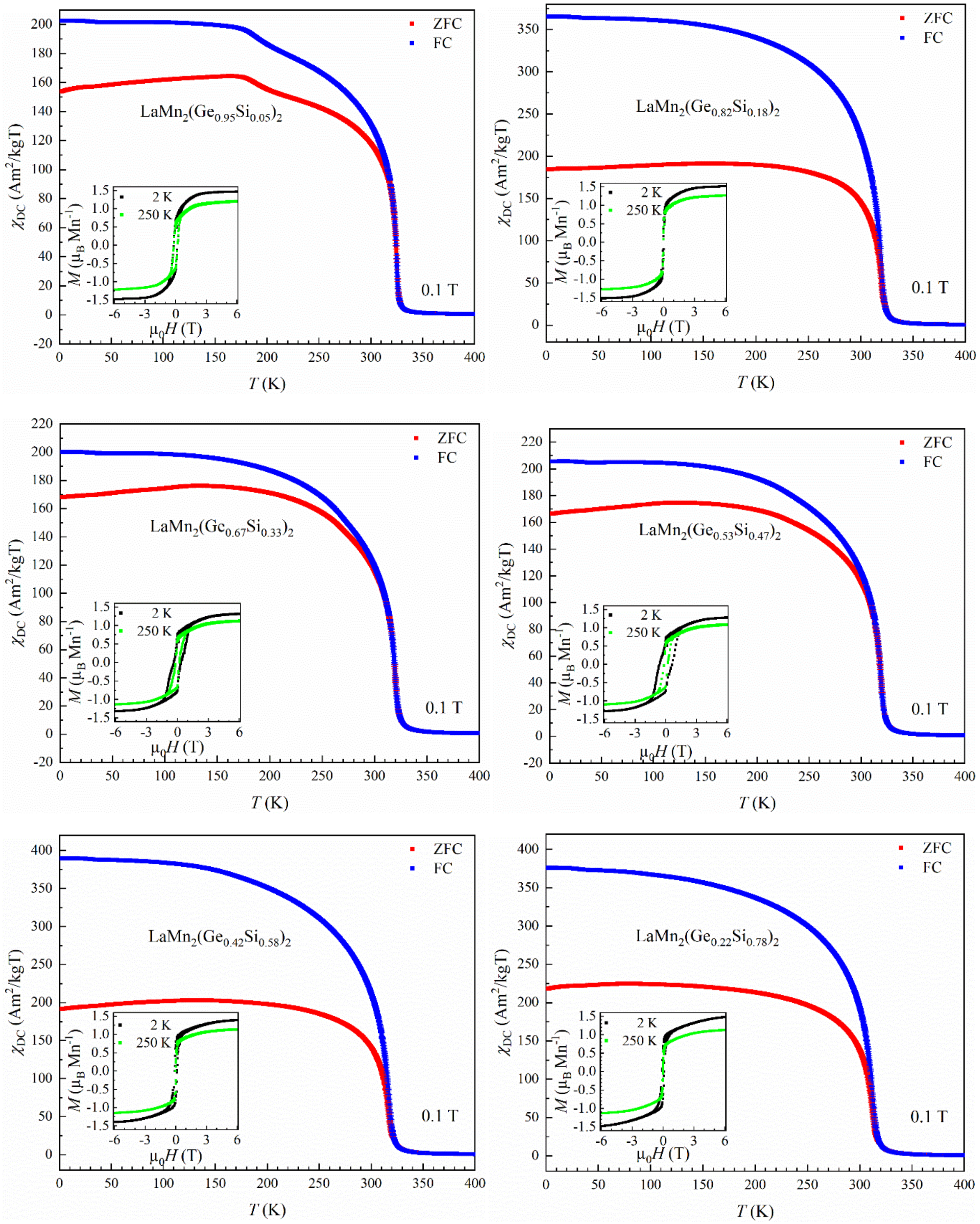

Figure S3: ZFC (*red*) and FC (*blue*) magnetic susceptibilities and isothermal magnetization (*insets*) of LaMn$_2$(Si$_{1\text{-}x}$Ge$_x$)$_2$ (x = 0.05, 0.18, 0.33, 0.47, 0.58, 0.78). The bump at approximately 180 K in LaMn$_2$(Ge$_{0.95}$ Si$_{0.05}$)$_2$ presumably originates from a minor ferromagnetic impurity as no magnetic phase changes were observed in the PND data. Its mass fraction, however, must be small as we could not identify any magnetic impurities from PXRD. All error bars are shown and represent 1σ. However, the error bars may be smaller than the symbol.

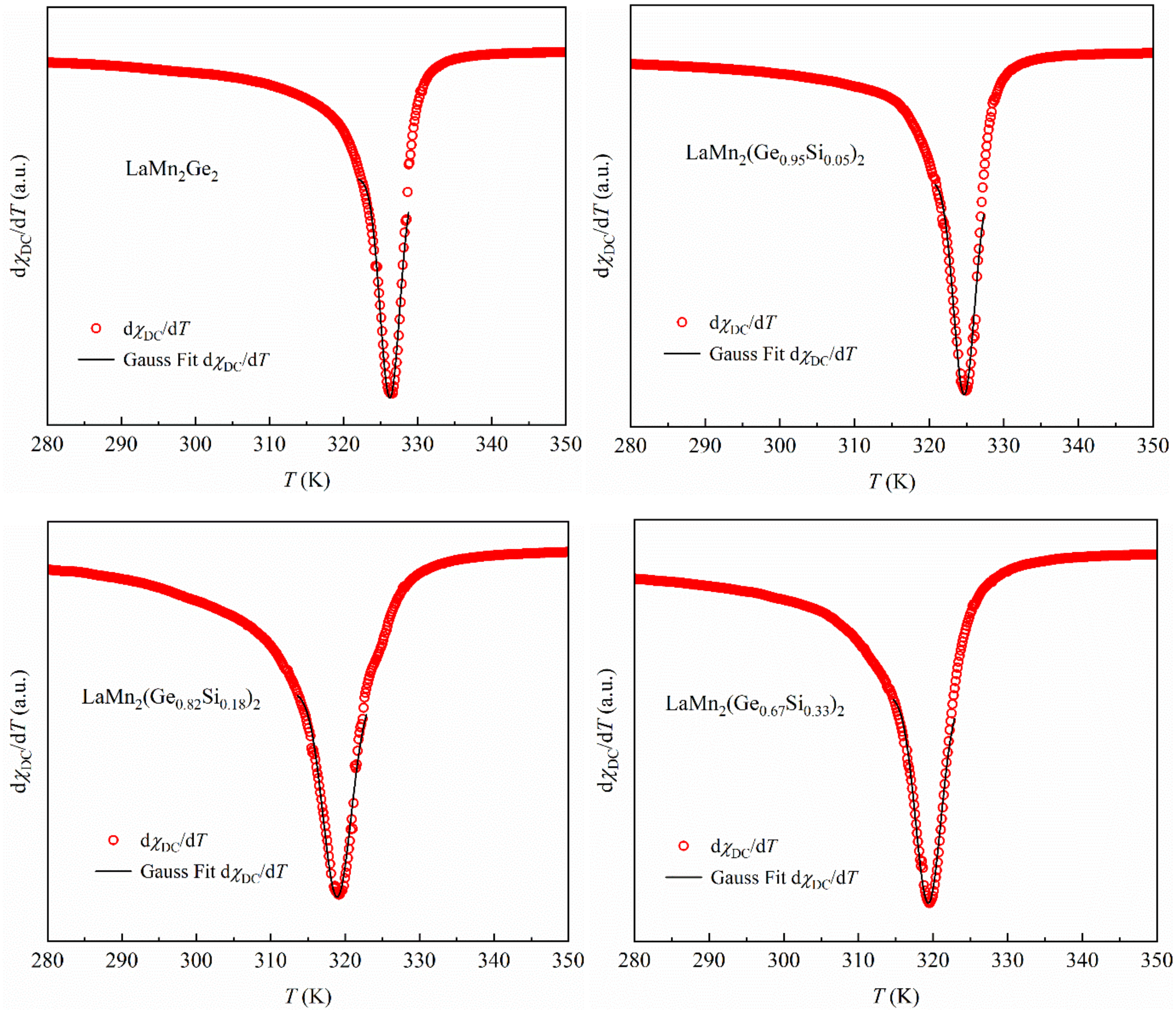

Figure S4a: First derivate of the DC magnetic susceptibility dχ_{DC}/dT of $LaMn_2(Si_{1-x}Ge_x)_2$ (x = 0, 0.05, 0.18, 0.33; *red*). The minimum was fitted with a Gaussian (*black*) to determine T_C. The data were smoothed before taking the derivative to avoid spikes. Thus, no error bars are shown.

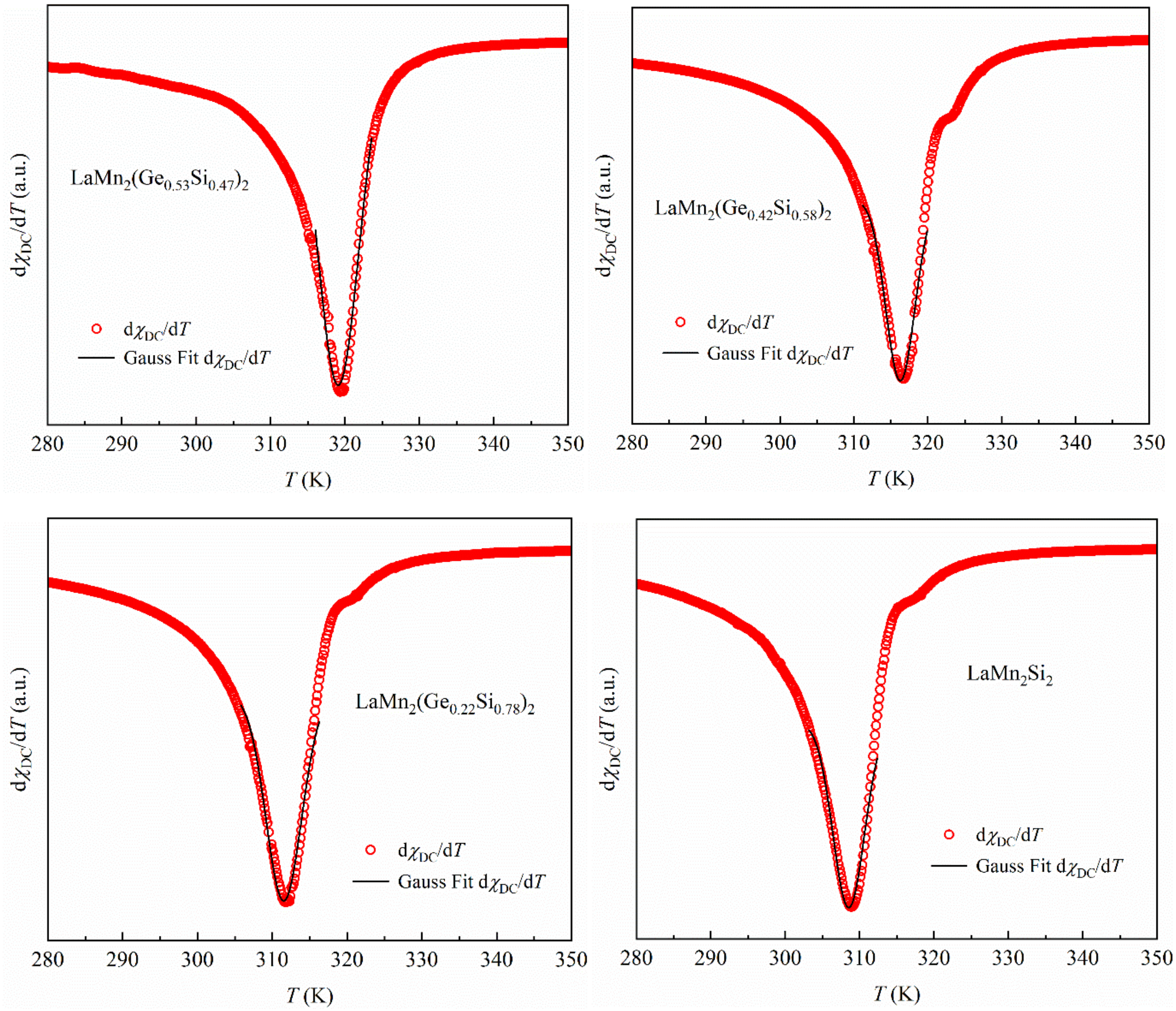

Figure S4b: First derivate of the DC magnetic susceptibility dχ_{DC}/dT of $LaMn_2(Si_{1-x}Ge_x)_2$ (x = 0.47, 0.58, 0.78, 1, *red*). The minimum was fitted with a Gaussian (*black*) to determine T_C. The data were smoothed before taking the derivative to avoid spikes. Thus, no error bars are shown.

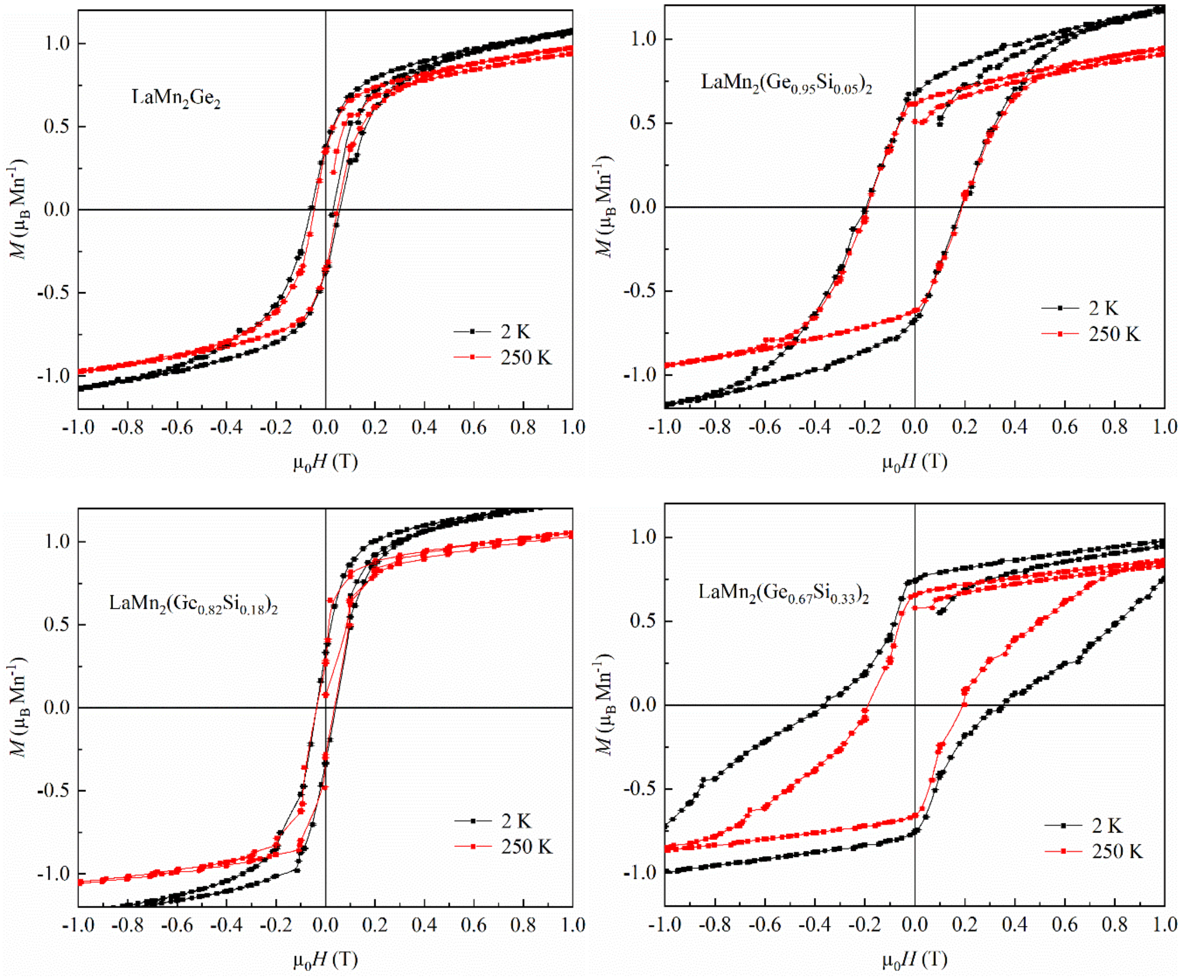

Figure S5a: Isothermal magnetization of $LaMn_2(Si_{1-x}Ge_x)_2$ (x = 0, 0.05, 0.18, 0.33) at 2 K (*black*) and 250 K (*red*) focusing on the hysteresis loop. All error bars are shown and represent 1σ. However, the error bars may be smaller than the symbol. The solid lines were added to guide the eye.

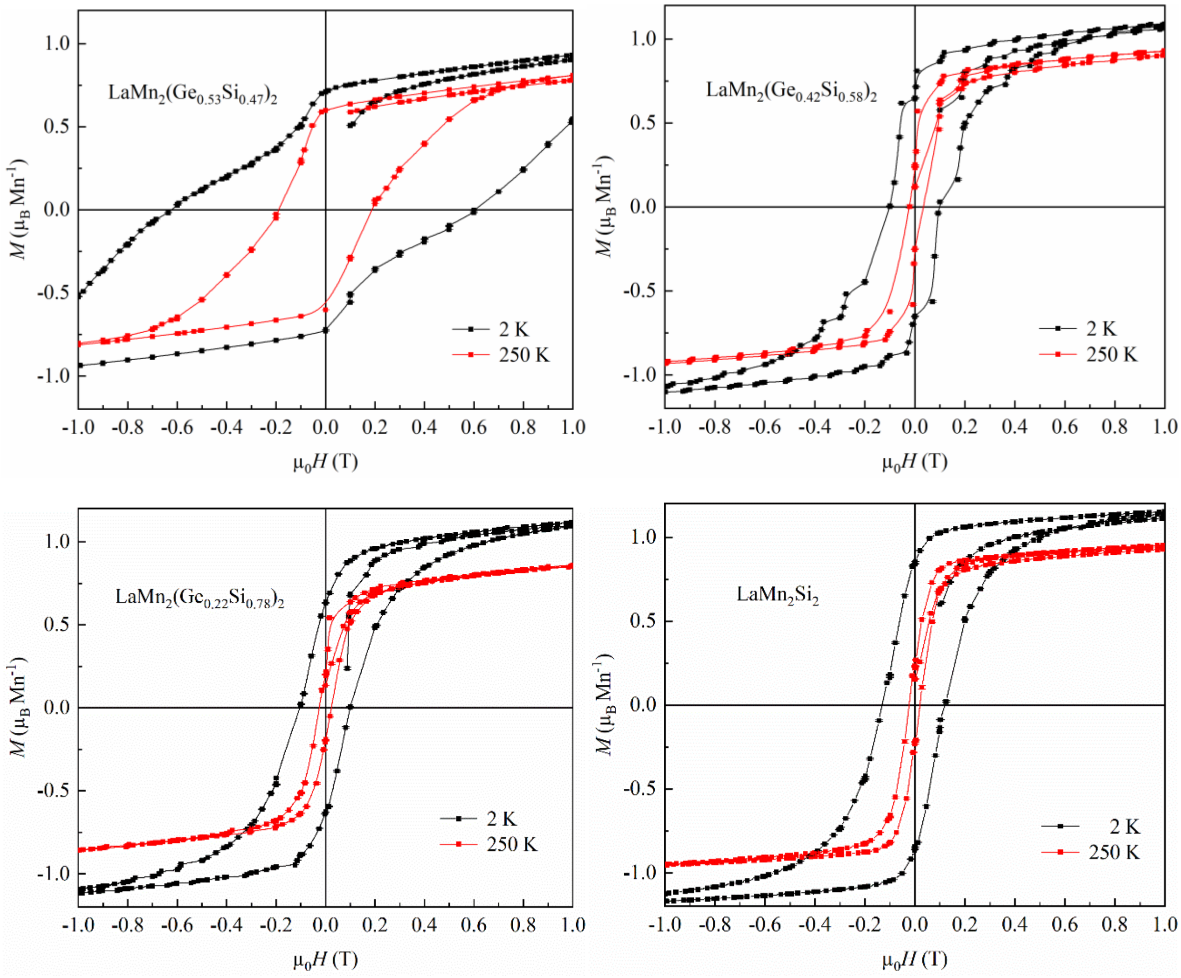

Figure S5b: Isothermal magnetization of $LaMn_2(Si_{1-x}Ge_x)_2$ (x = 0.47, 0.58, 0.78, 1) at 2 K (*black*) and 250 K (*red*) focusing on the hysteresis loop. All error bars are shown and represent 1σ. However, the error bars may be smaller than the symbol. The solid lines were added to guide the eye.

Table S1: Coercive fields *H*c of $LaMn_2(Ge_{1-x}Si_x)_2$ (x = 0, 0.05, 0.18, 0.33, 0.47, 0.58, 0.78, 1) at 2 K and 250 K. *H*c was estimated from the isothermal magnetization using linear fits. The numbers between parentheses show the error bars and represent 1σ.

Compound (at.%)	*H*c (mT) at 2 K	*H*c (mT) at 250 K
x = 0	59(1)	48(1)
x = 0.05	196(1)	188(7)
x = 0.18	39(1)	30(1)
x = 0.33	356(9)	173(1)
x = 0.47	620(7)	203(9)
x = 0.58	108(6)	27(1)
x = 0.78	105(3)	27(1)
x = 1	127(1)	24(1)

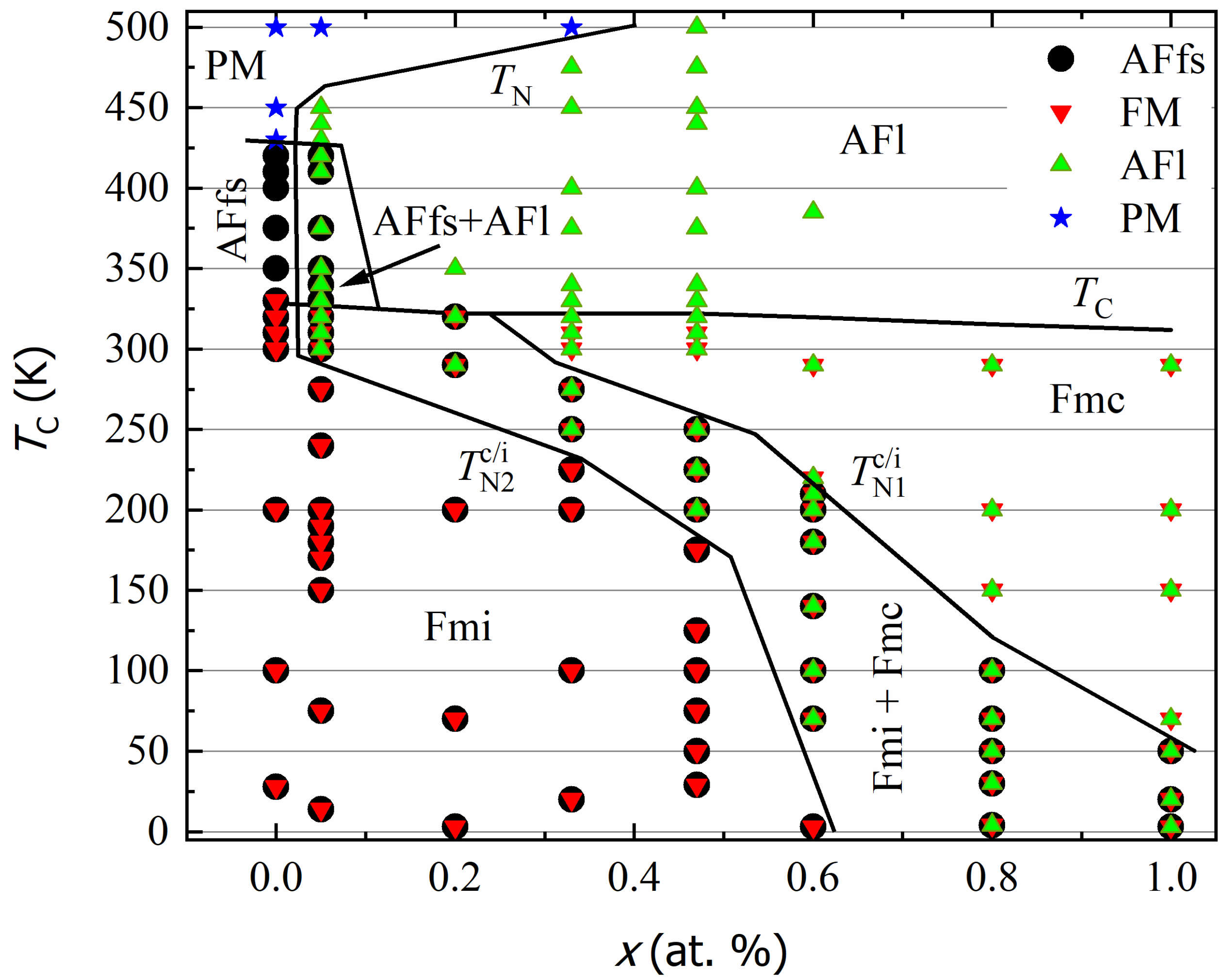

Figure S6: *x*-*T* magnetic phase diagram of the solid solution $LaMn_2(Ge_{1-x}Si_x)_2$ showing all measured temperatures points. The symbols refer to the different magnetic components, the areas to the various magnetic phases: Paramagnetic (PM), antiferromagnetic flat spiral (AFfs), antiferromagnetic layers (AFl), ferromagnetic mixed incommensurate (Fmi), ferromagnetic mixed commensurate (Fmc).

References

1 Müller-Buschbaum, H. & Schnering, H. G. V. Zur Struktur der A-Form der Sesquioxide der Seltenen Erden. Strukturuntersuchungen an La2O3. *Z. Anorg. Allg. Chem.* **340**, 232-245, https://doi.org/10.1002/zaac.19653400503 (1965).

2 Yusupov, R. G. *et al.* Mavlyanovite, Mn5Si3: a new mineral species from a lamproite diatreme, Chatkal Ridge, Uzbekistan. *Mineral. Mag.* **73**, 43-50, https://doi.org/10.1180/minmag.2009.073.1.43 (2009).

3 Masubuchi, Y., Higuchi, M., Takeda, T. & Kikkawa, S. Oxide ion conduction mechanism in RE9.33(SiO4)6O2 and Sr2RE8(SiO4)6O2 (RE=La, Nd) from neutron powder diffraction. *Solid State Ionics* **177**, 263-268, https://doi.org/10.1016/j.ssi.2005.09.015 (2006).
